# Supplementary material for: Impact of protein and small molecule interactions on kinase conformations
Source: eLife. 2024 Aug 1;13:RP94755. doi: 10.7554/eLife.94755 (PMC11293870; doi:10.7554/eLife.94755)
Supplement: Figure 1—source data 2. [file elife-94755-fig1-data2.pdf]

Indicated antibodies have been used (for details see the Materials and Methods section)

Figure 1 Panel E: KinCon-time course experiments:

In these panels western blots are shown. The corresponding western blot raw data is enclosed below:

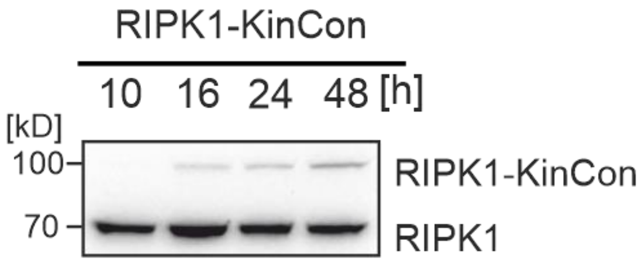

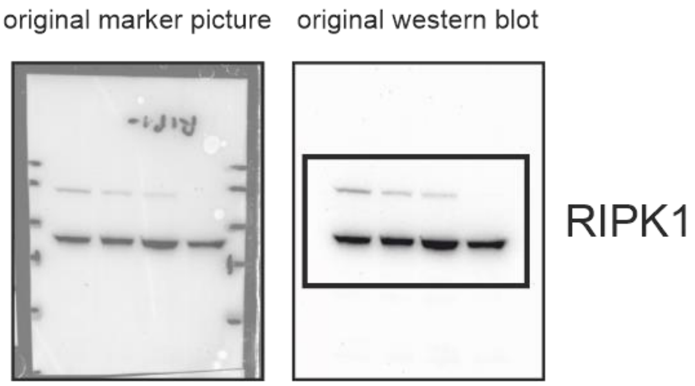

Blots were mirrored for figure

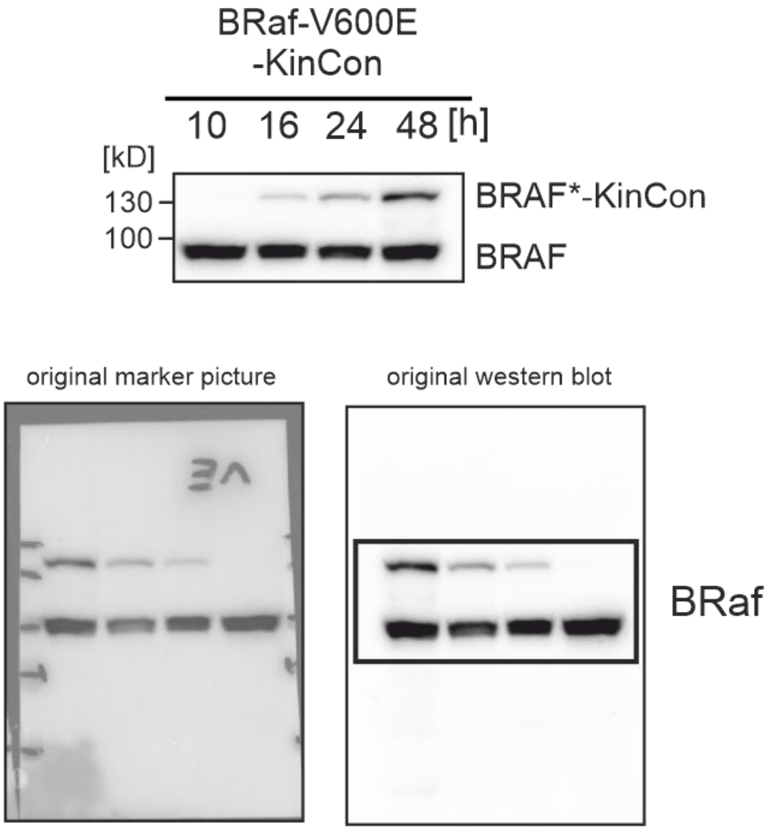

Blots were mirrored for figure

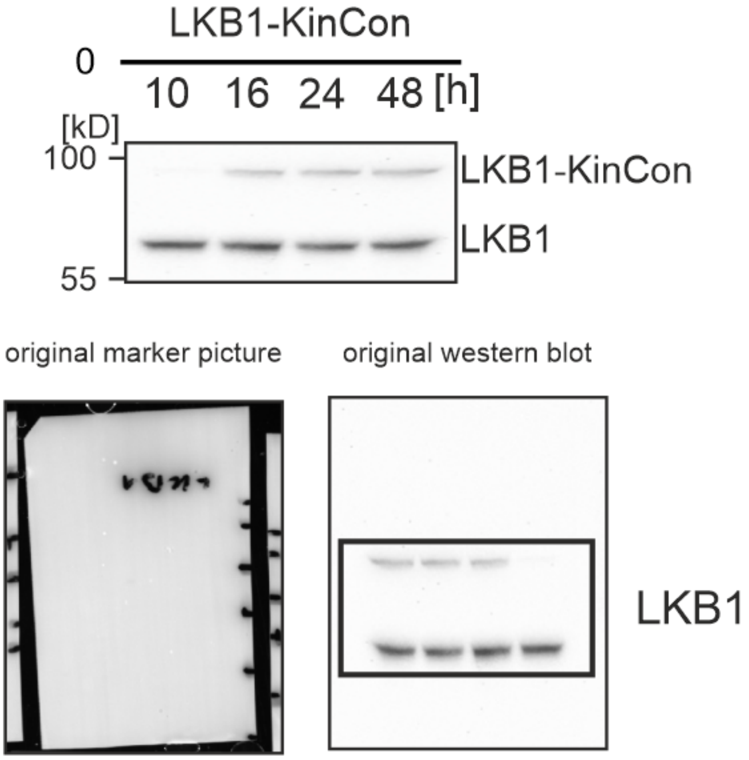

Blots were mirrored for figure

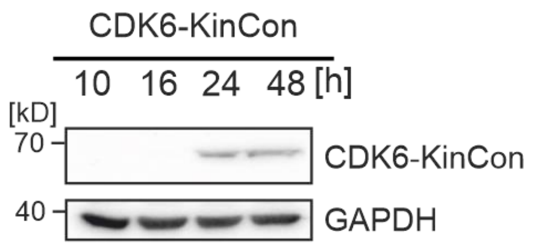

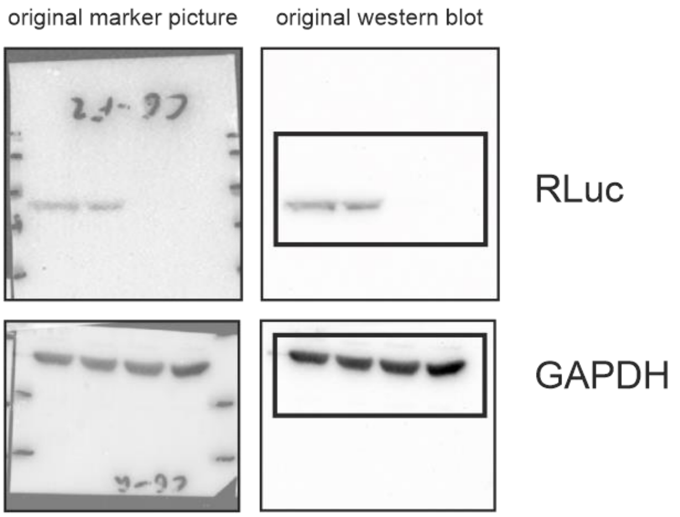

Blots were mirrored for figure

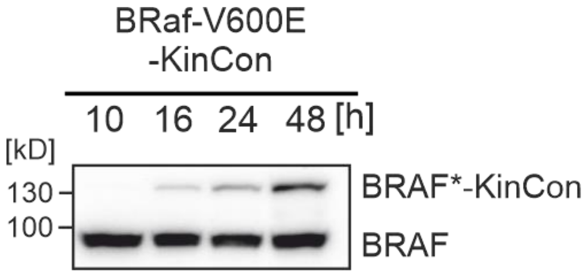

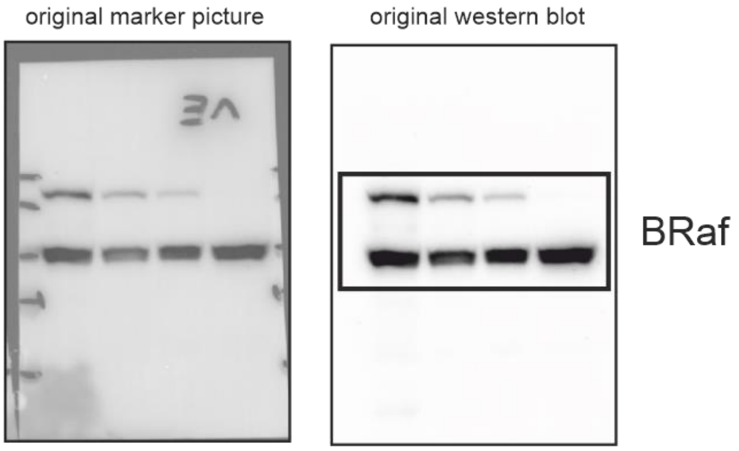

Blots were mirrored for figure

Figure 1 Panel F: BRaf-PLX-KinCon-time course experiments:

In these panels western blots are shown. The corresponding western blot raw data is enclosed blow :

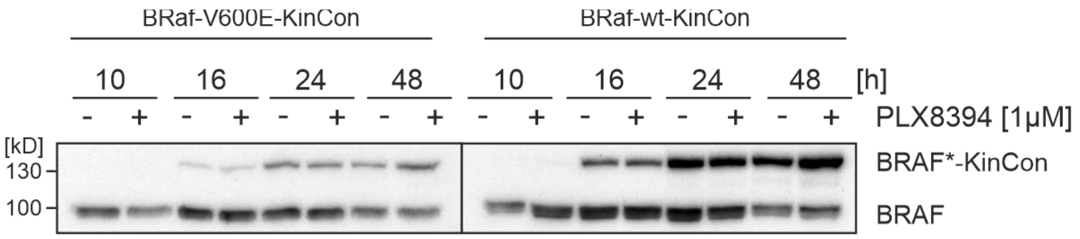

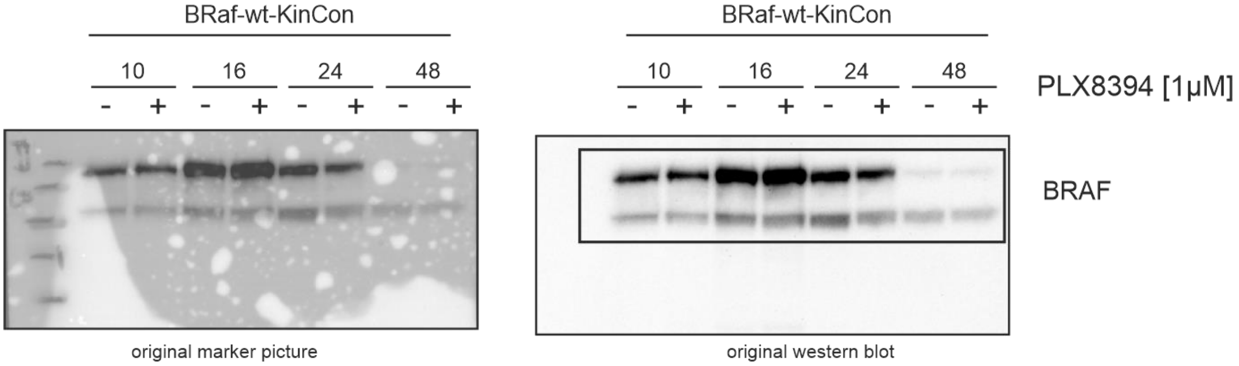

Blots were mirrored for figure

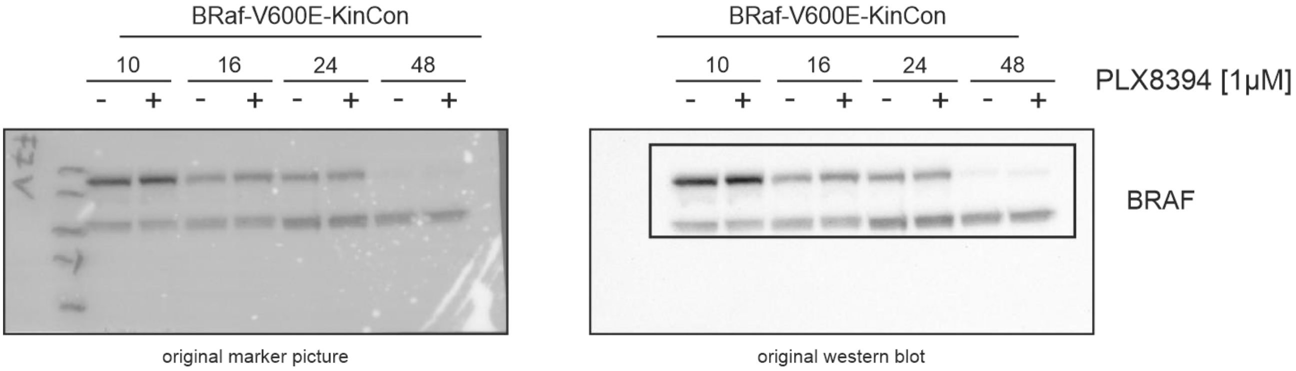

Blots were mirrored for figure
