# Supplementary material for: Impact of protein and small molecule interactions on kinase conformations
Source: eLife. 2024 Aug 1;13:RP94755. doi: 10.7554/eLife.94755 (PMC11293870; doi:10.7554/eLife.94755)
Supplement: Figure 2—source data 2. [file elife-94755-fig2-data2.pdf]

Indicated antibodies have been used (for details see the Materials and Methods section)

Figure 2 Panel D: Impact of LKB1-STRADa-MO25 co-expression of pAMPK/AMPK phosphorylation pattern.

In this panel a representative western blot is shown. The corresponding western blot raw data is enclosed blow:

Replicate 1:

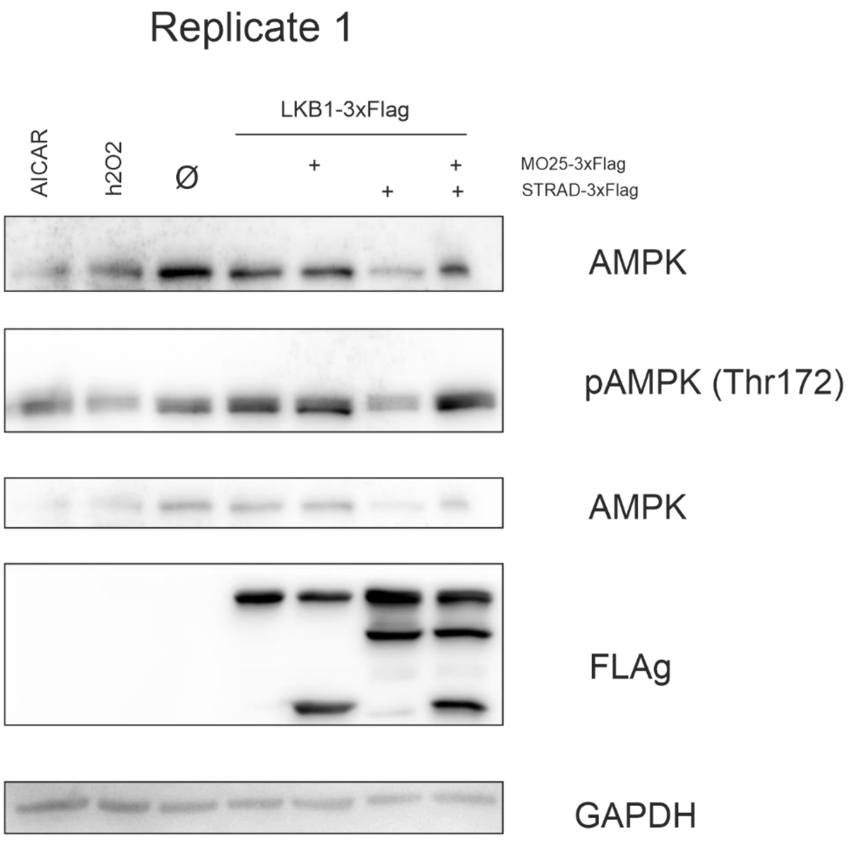

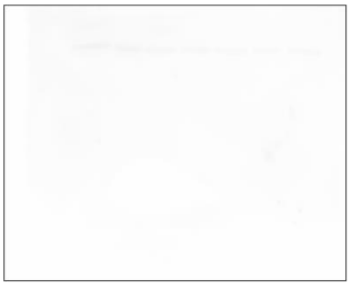

original western blot
GAPDH

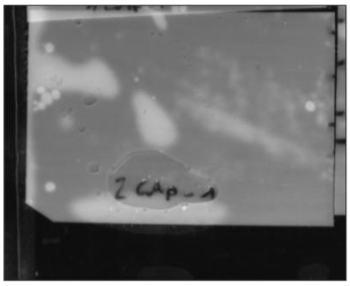

original marker picture
GAPDH

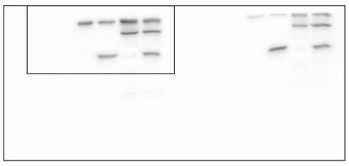

original western blot

Flag

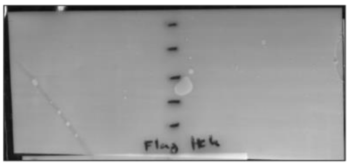

original marker picture
Flag

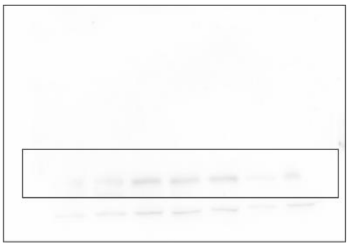

original western blot

AMPK

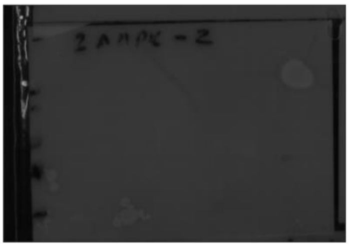

original marker picture

AMPK

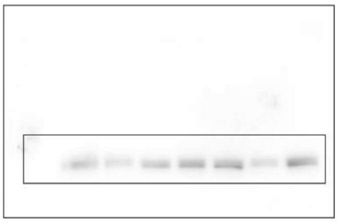

original marker picture

pAMPK (Thr172)

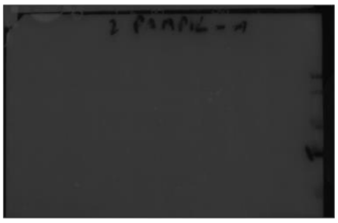

original western blot
pAMPK (Thr172)

Replicate 2:

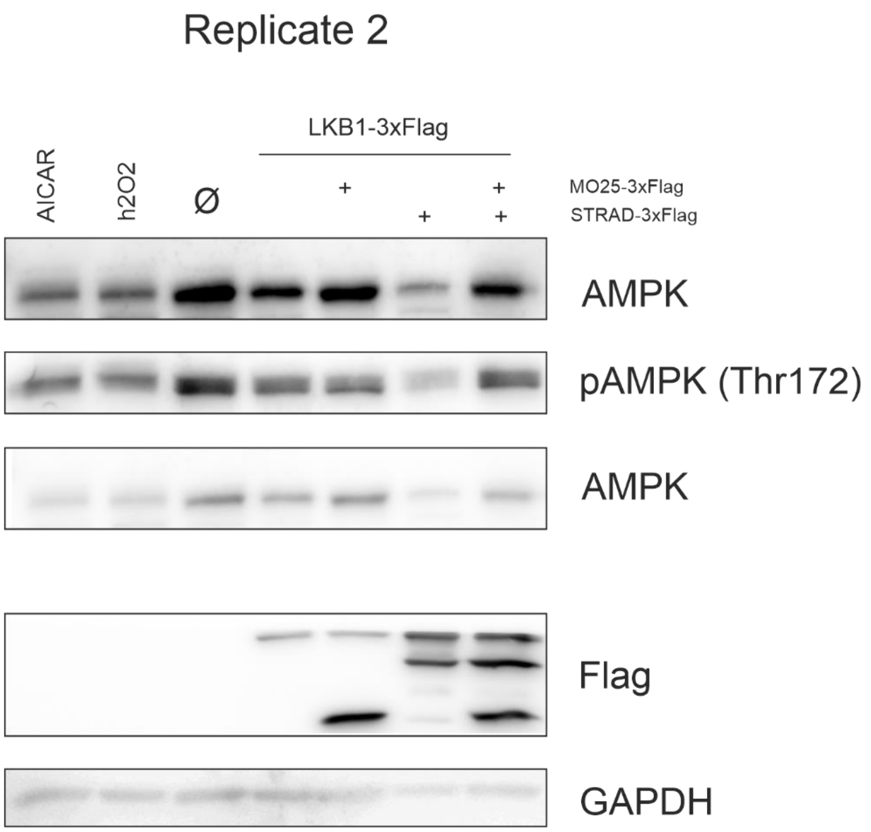
Replicate 2
LKB1-3xFlag
AICAR
h2O2
Ø
+
+
+
+
MO25-3xFlag
STRAD-3xFlag
AMPK
pAMPK (Thr172)
AMPK
Flag
GAPDH

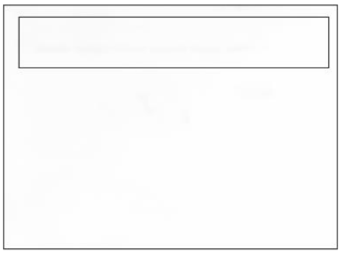

original western blot
GAPDH

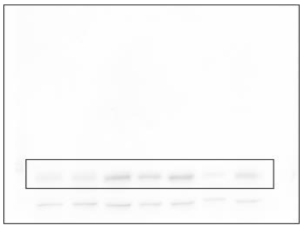

original western blot

AMPK

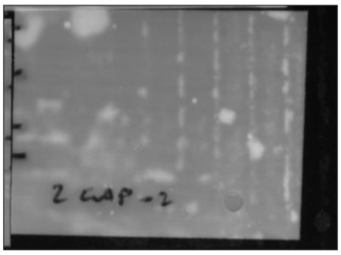

original marker picture

GAPDH

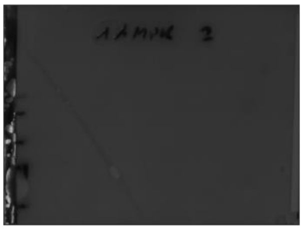

original marker picture

AMPK

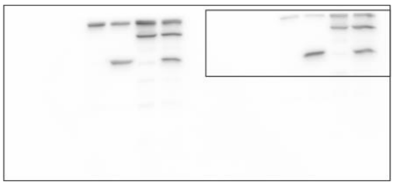

original western blot

Flag

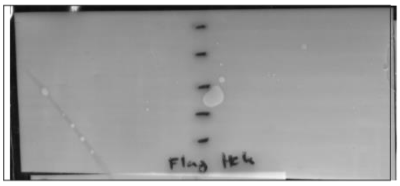

original marker picture

Flag

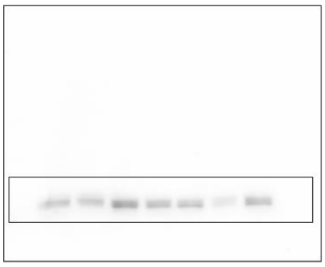

original western blot

pAMPK (Thr172)

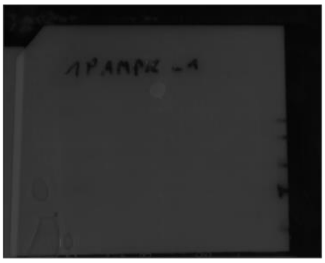

original marker picture

pAMPK (Thr172)

Replicate 3:

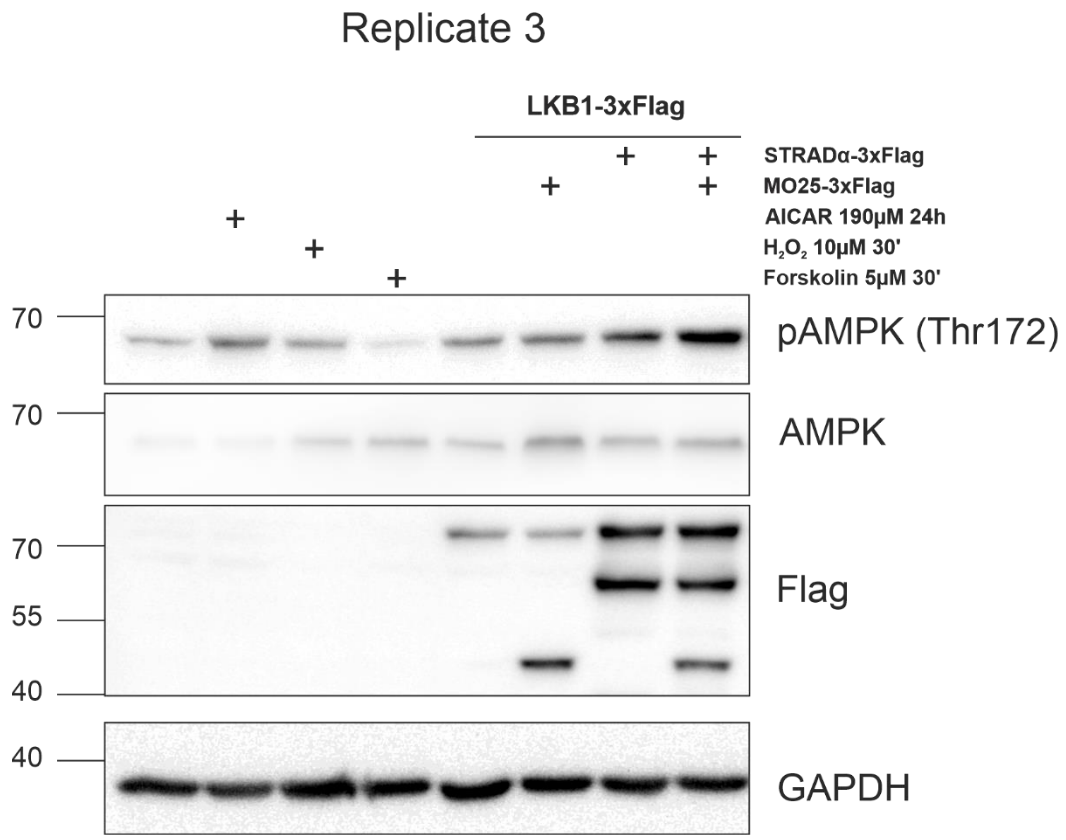

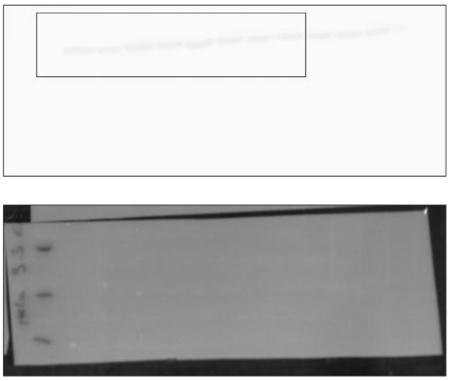

original western blot
GAPDH

original marker picture

GAPDH

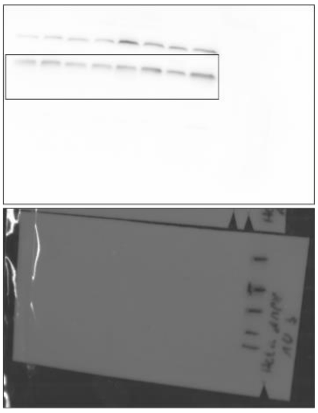

original western blot

AMPK

original marker picture

AMPK

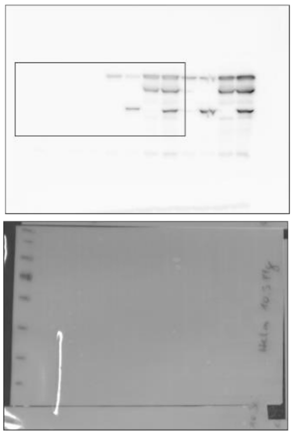

original western blot

Flag

original marker picture

Flag

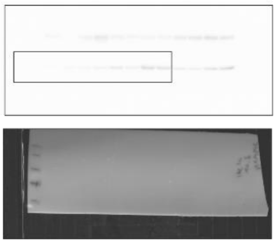

original western blot

pAMPK (Thr172)

original marker picture

pAMPK (Thr172)

Replicate 4:

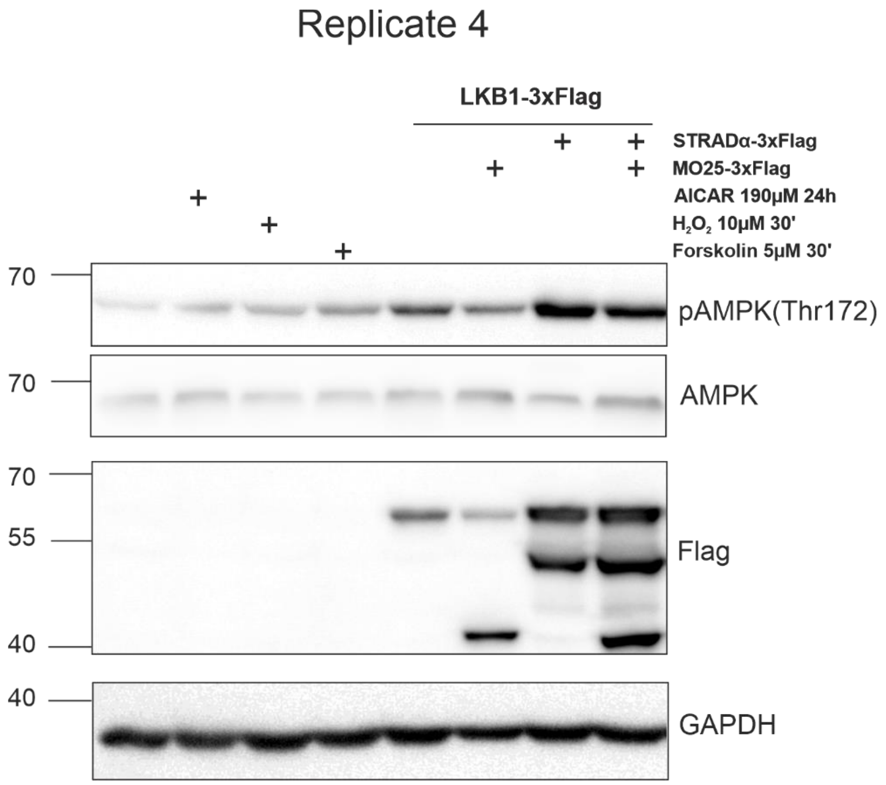
Replicate 4
LKB1-3xFlag
STRADα-3xFlag
MO25-3xFlag
AICAR 190μM 24h
H2O2 10μM 30'
Forskolin 5μM 30'
70
pAMPK(Thr172)
70
AMPK
70
55
40
Flag
40
GAPDH

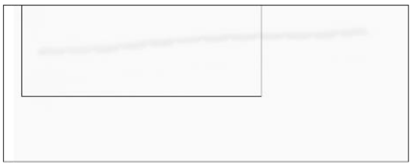

original western blot

GAPDH

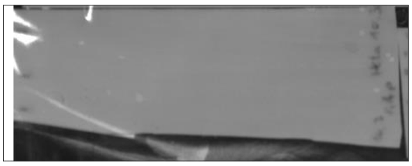

original marker picture

GAPDH

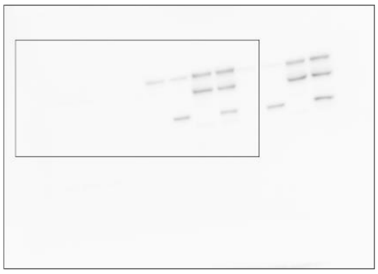

original western blot

Flag

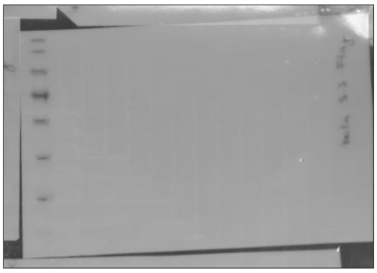

original marker picture

Flag

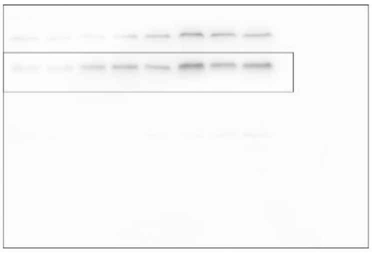

original western blot

AMPK

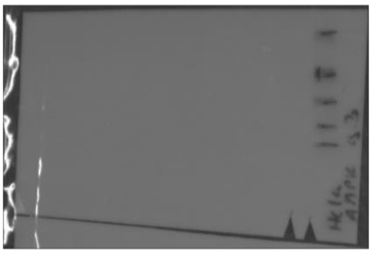

original marker picture

AMPK

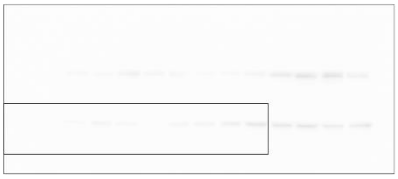

original western blot

pAMPK (Thr172)

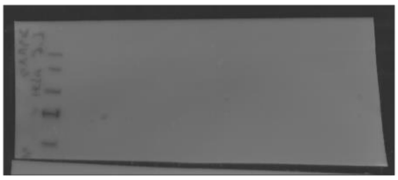

original marker picture

pAMPK (Thr172)

Figure 2 Panel E/F: Impact of LKB1-STRADa-MO25 co-expression on STRADa/LKB1 conformation

In these panels representative western blots are shown. Since they were often detected on the same membranes panel E and F are shown here together. The corresponding western blot raw data is enclosed below :

Replicate 1F,1E, 2F:

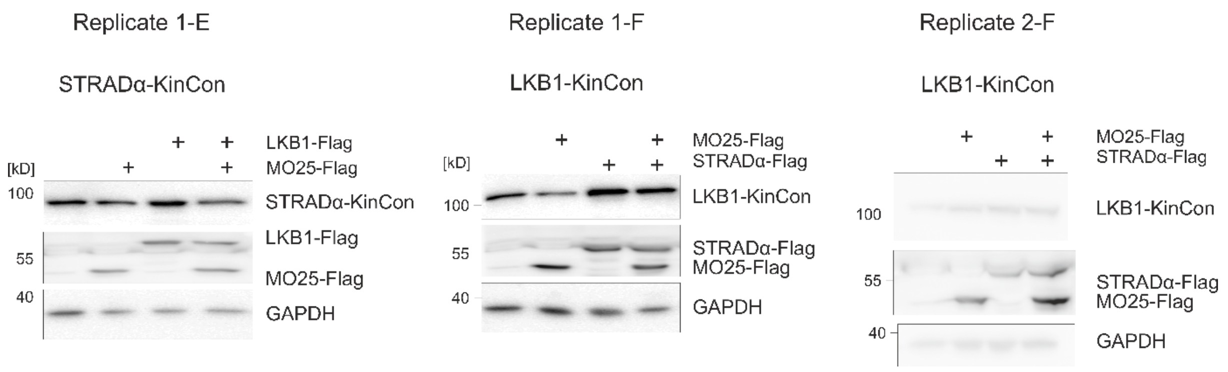

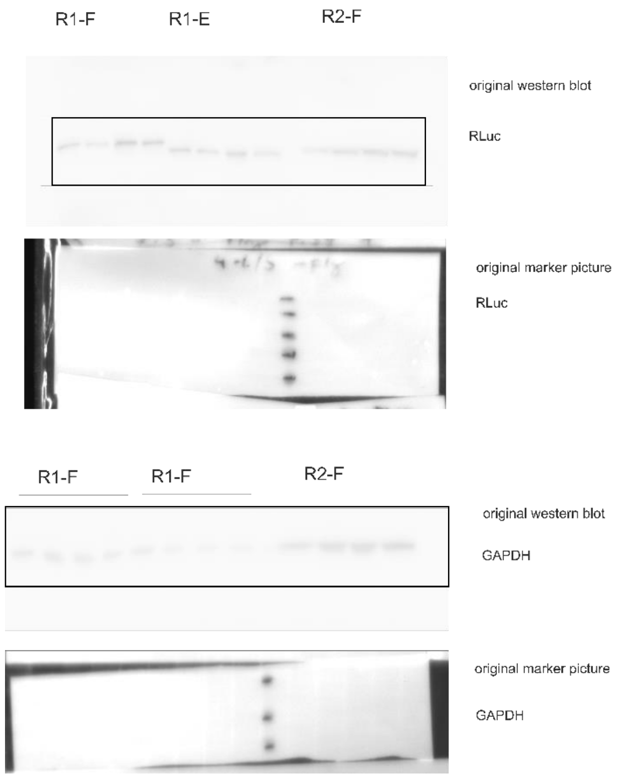

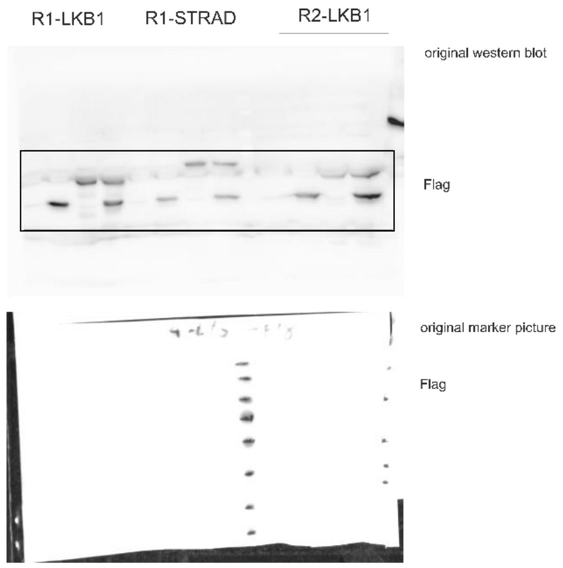

Replicate 3F,2E:

Replicate 2-E

STRADα-KinCon

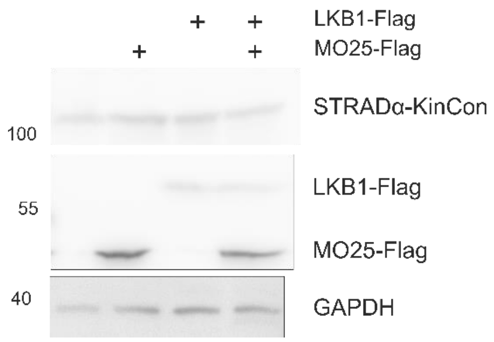

Replicate 3-F

LKB1-KinCon

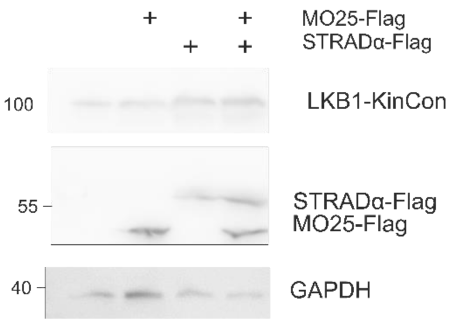

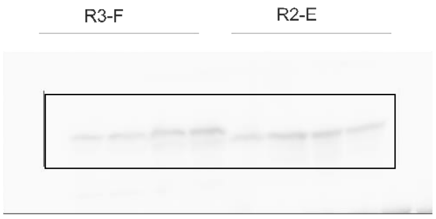

original western blot

RLuc

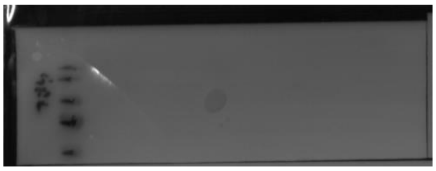

original marker picture

RLuc

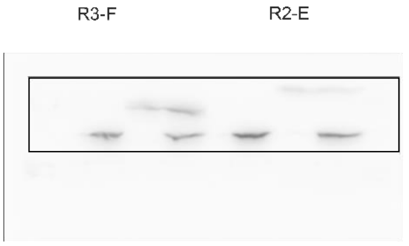

original western blot

Flag

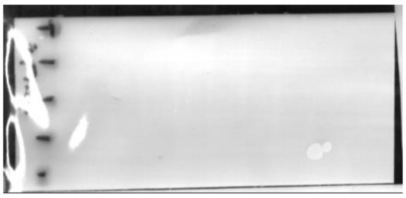

original marker picture

Flag

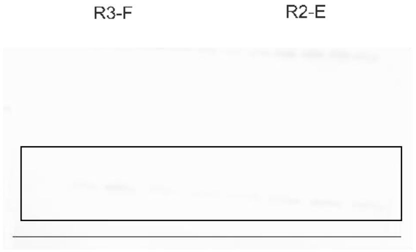

original western blot

GAPDH

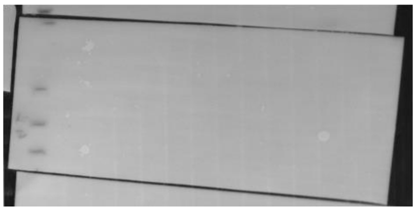

original marker picture

GAPDH

Replicate 3E:

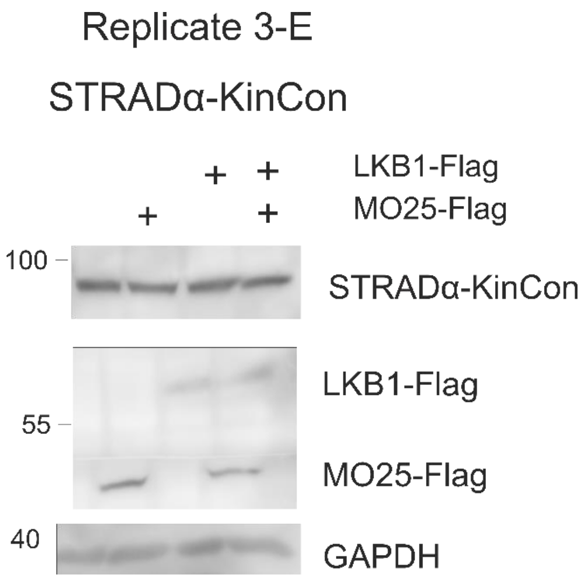

R3-E

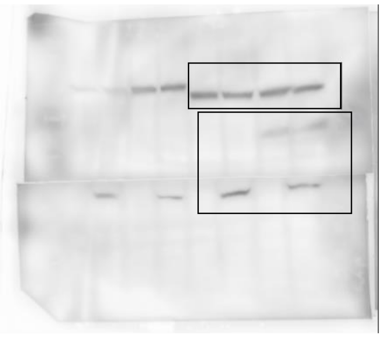

original western blot

RLuc

Flag

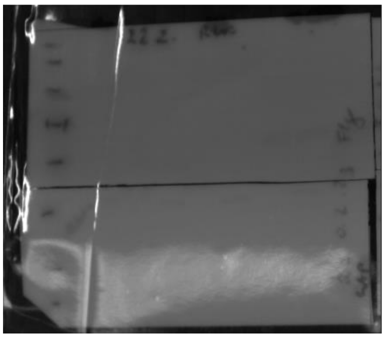

original marker picture

RLuc + Flag

R3-E

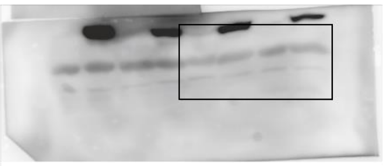

original western blot

GAPDH

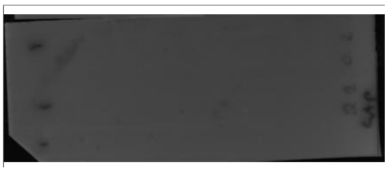

original marker picture

GAPDH

Replicate 4F,4E:

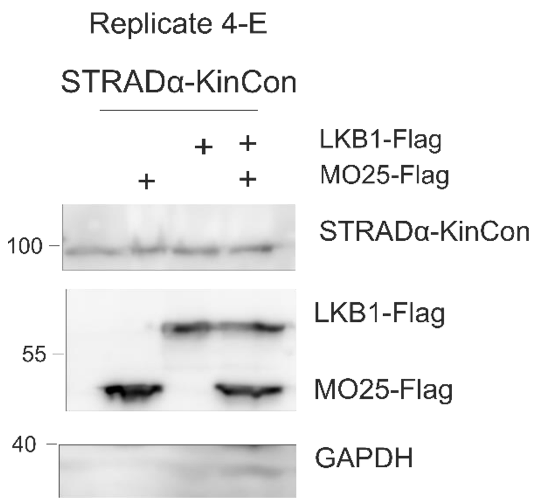

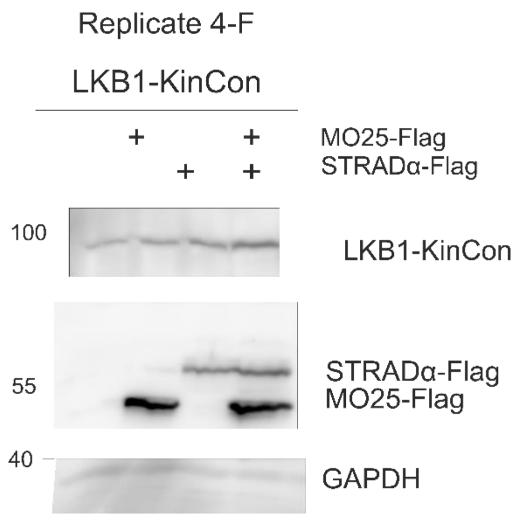

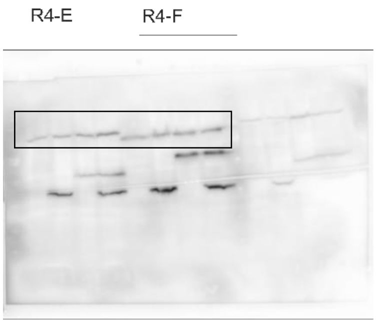

original western blot

RLuc

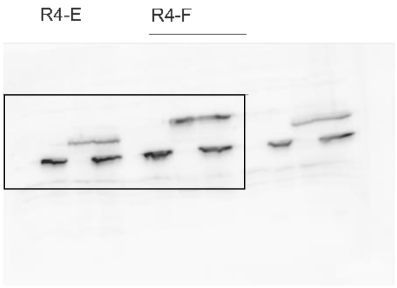

original western blot

Flag

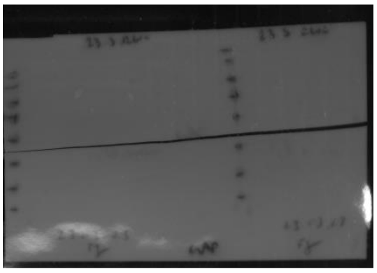

original marker picture

RLuc

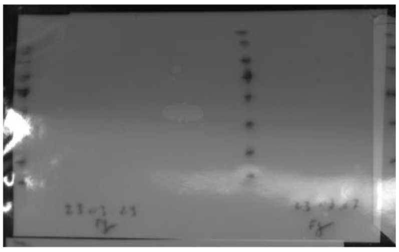

original marker picture

Flag

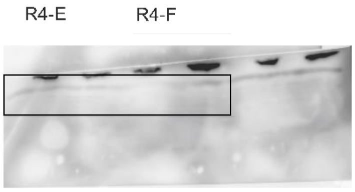

original western blot

GAPDH

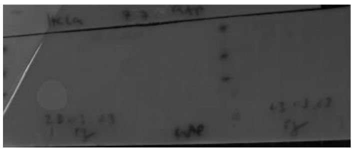

original marker picture

GAPDH

Replicate 5 -Figure E:

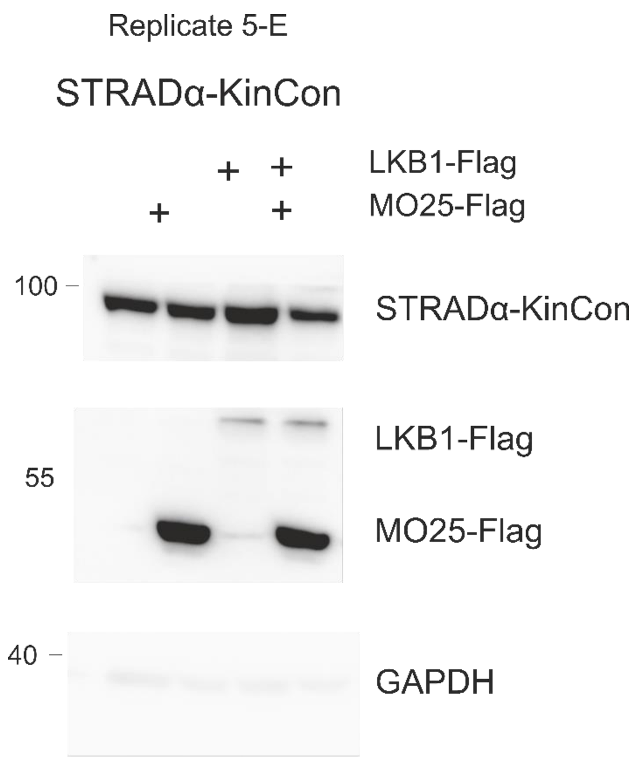

R5-E

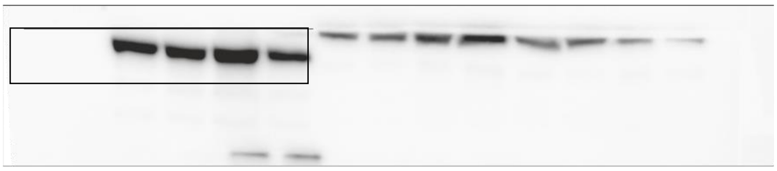

original western blot
RLuc

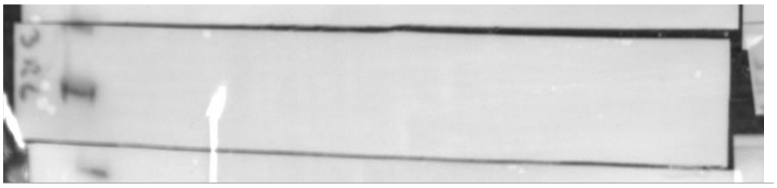

original marker picture

RLuc

R5-E

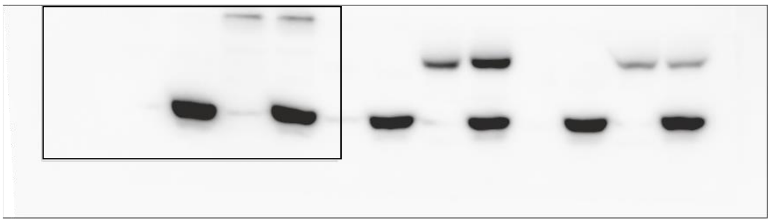

original western blot
Flag

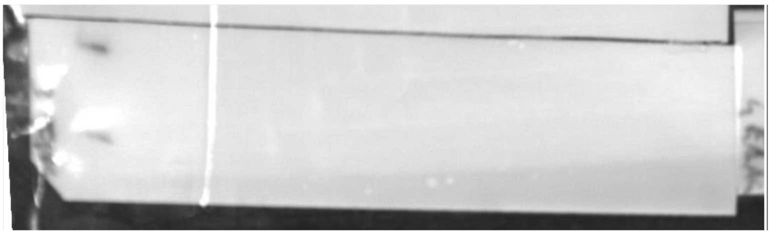

original marker picture

Flag

R5-E

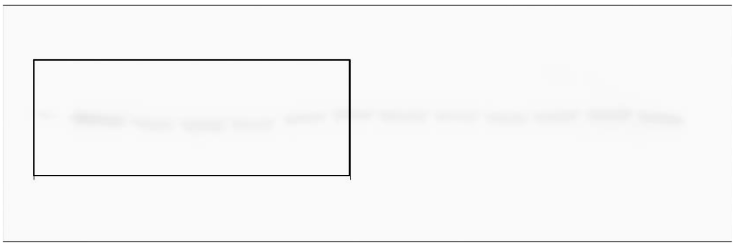

original western blot

GAPDH

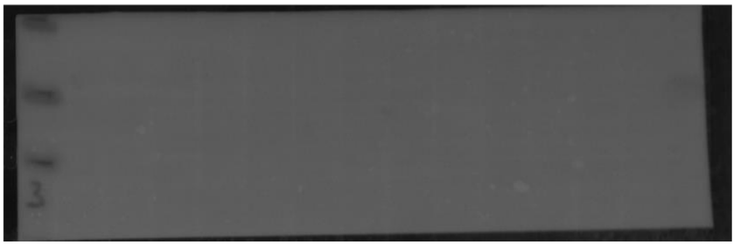

original marker picture

GAPDH

Figure 2 Panel G: Impact of STRADa-H231/F223A coexpression on LKB1 conformation

In these panels representative western blots are shown. The corresponding western blot raw data is enclosed below and:

Replicate 1:

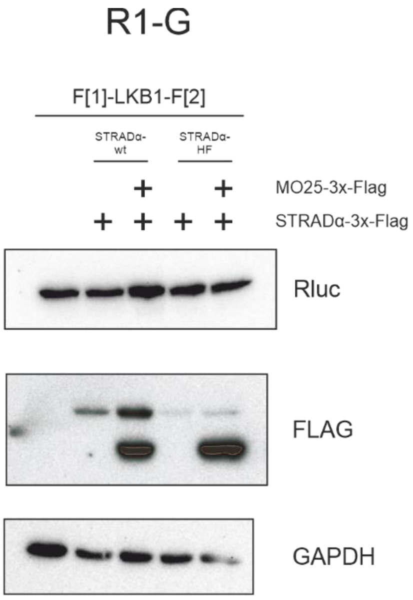

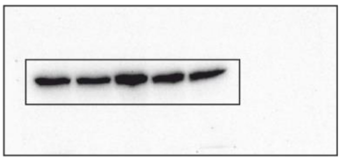

original western blot

RLuc

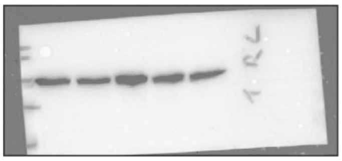

original marker picture

RLuc

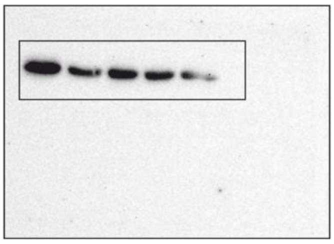

original western blot

GAPDH

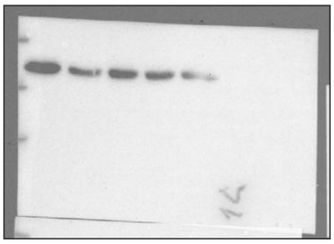

original marker picture

GAPDH

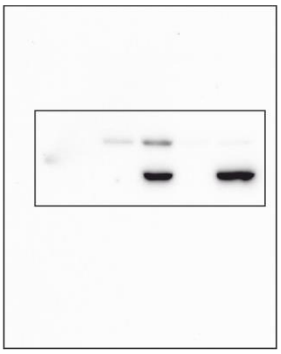

original western blot

Flag

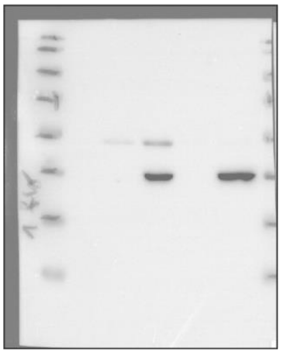

original marker picture

Flag

Replicate 2:

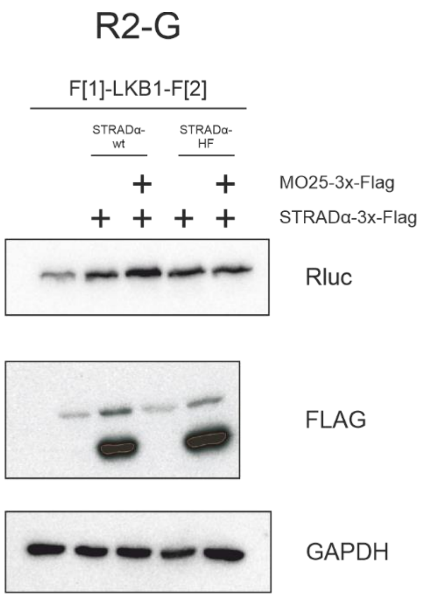

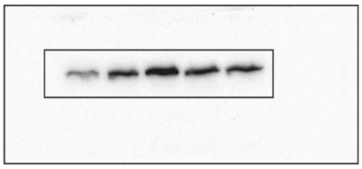

original western blot

RLuc

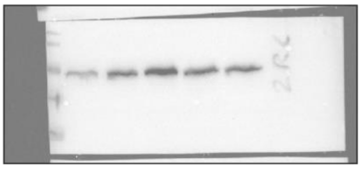

original marker picture

RLuc

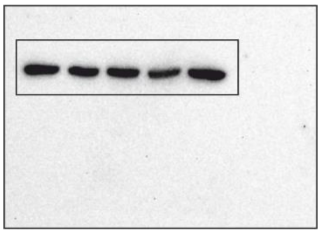

original western blot

GAPDH

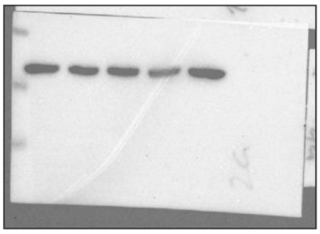

original marker picture

GAPDH

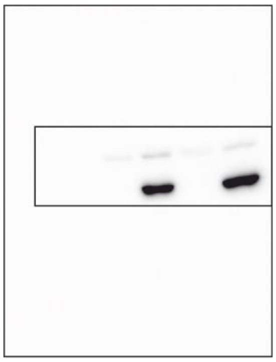

original western blot

Flag

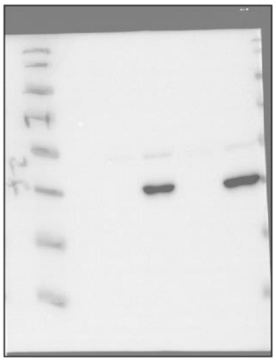

original marker picture

Flag

Replicate 3:

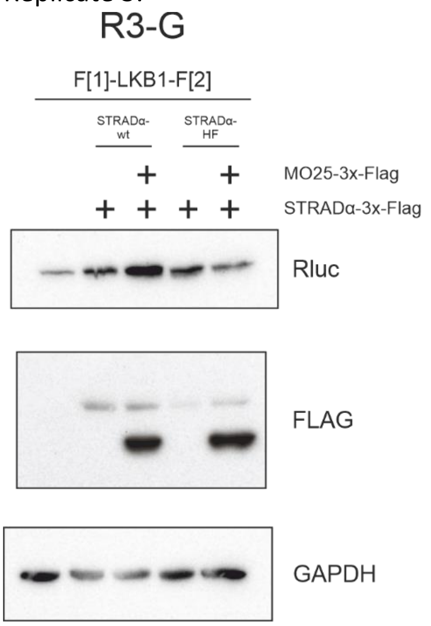

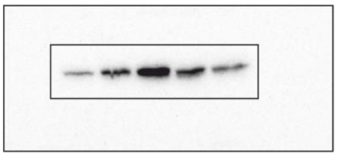

original western blot

RLuc

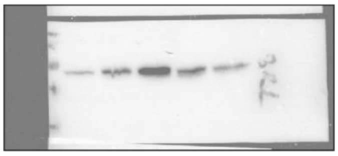

original marker picture

RLuc

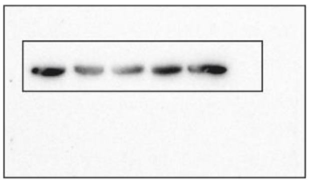

original western blot

GAPDH

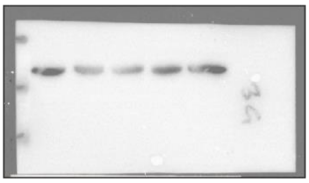

original marker picture

GAPDH

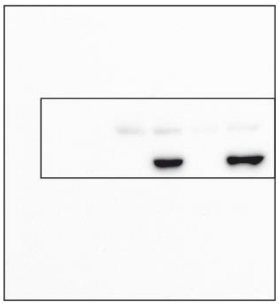

original western blot

Flag

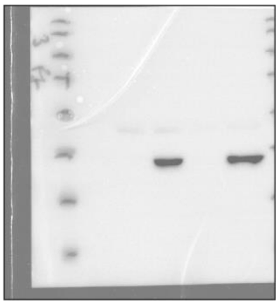

original marker picture

Flag

Replicate 4:

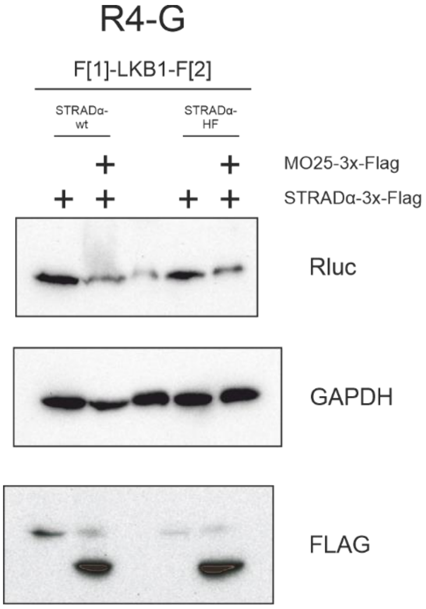

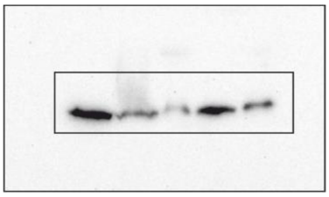

original western blot

RLuc

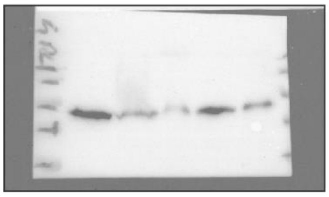

original marker picture

RLuc

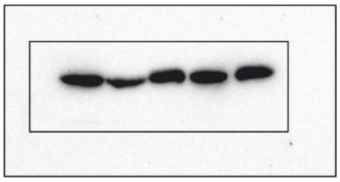

original western blot

GAPDH

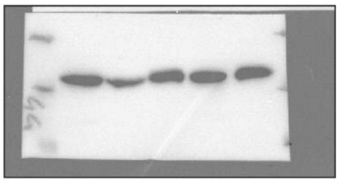

original marker picture

GAPDH

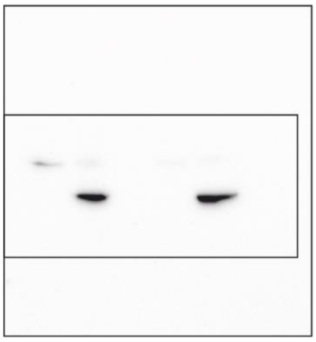

original western blot

Flag

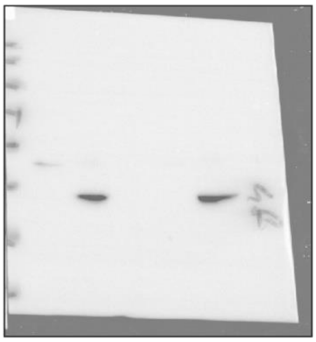

original marker picture

Flag

Figure 2 Panel I: Impact of LKB1-mutants on the LKB1 conformation:

In these panels representative western blots are shown. The corresponding western blot raw data is enclosed below :

Replicate1:

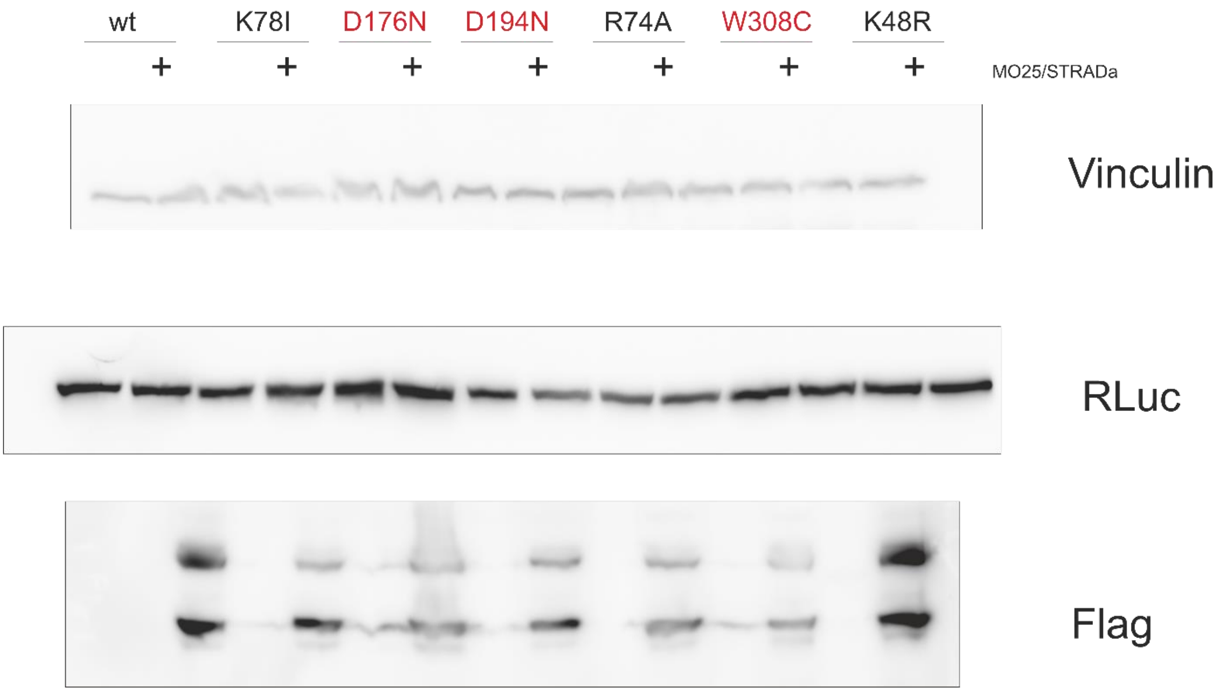

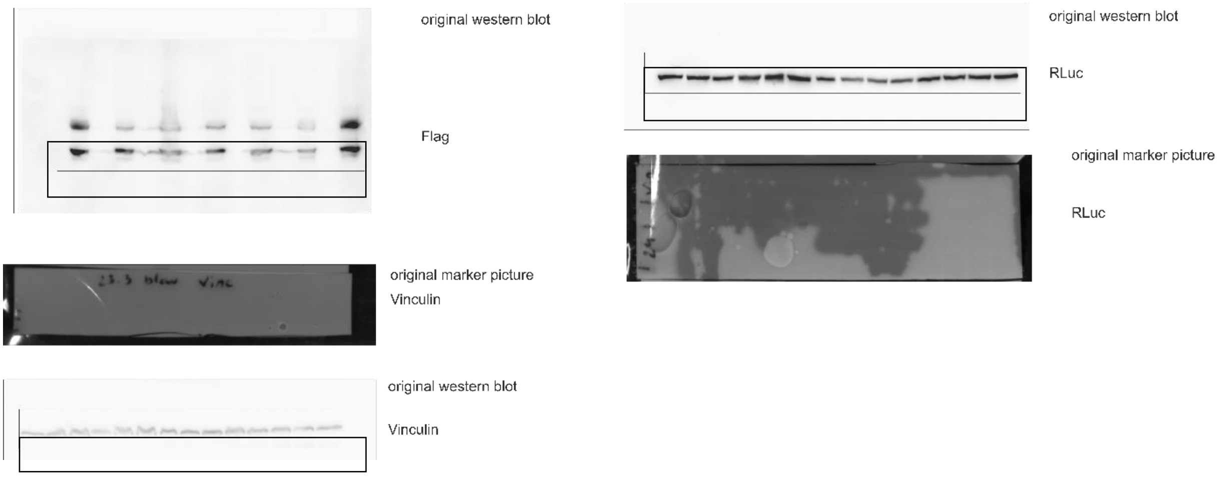

For this replicate no marker picture was detected in the case of the Flag antibody

Replicate 2:

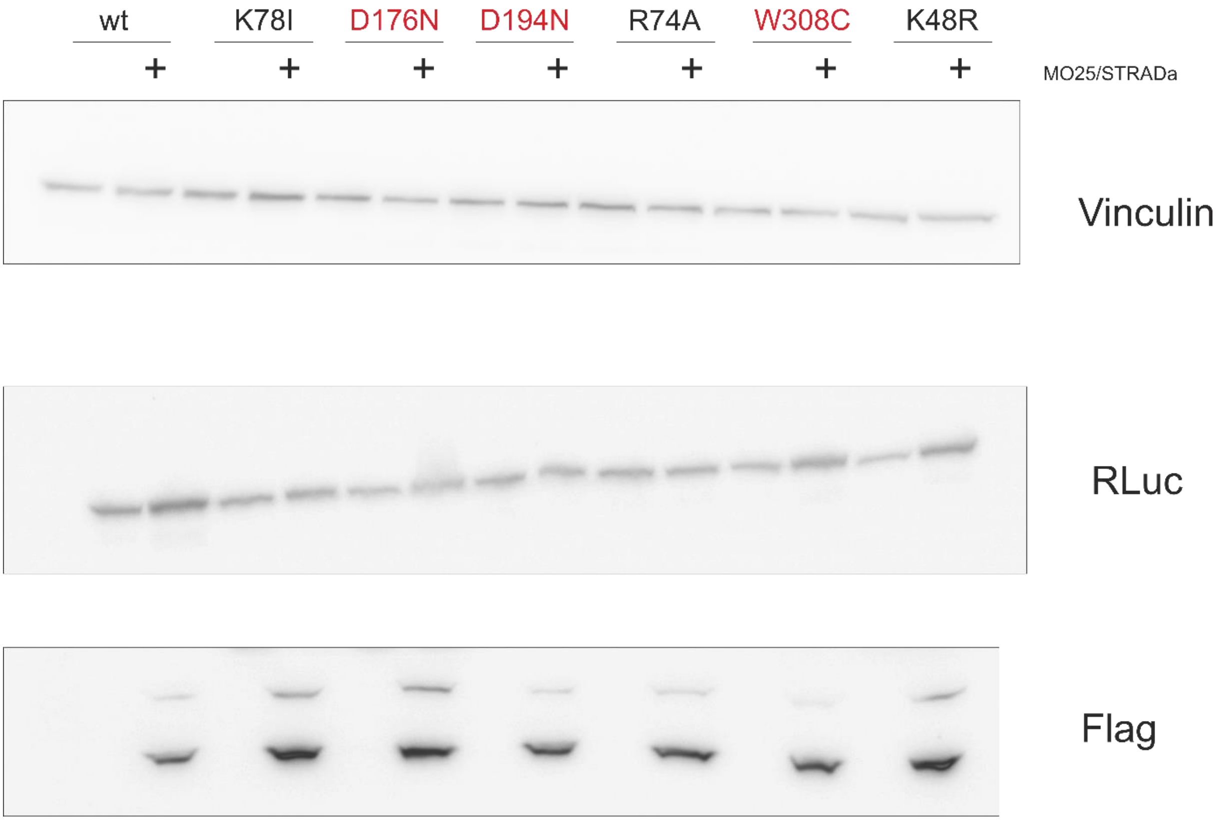

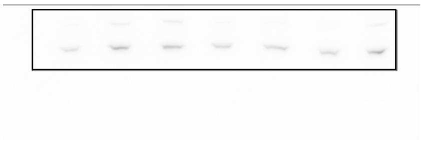

original western blot
Flag

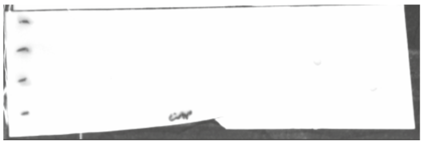

original marker picture
Flag

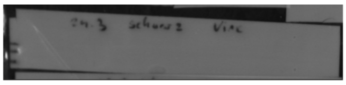

original marker picture
Vinculin

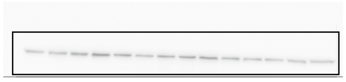

original western blot
Vinculin

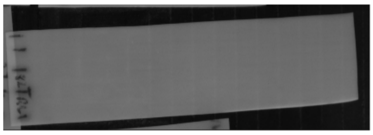

original marker picture
RLuc

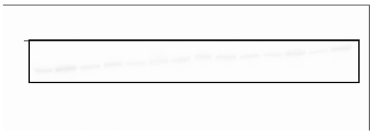

original western blot
RLuc

Replicate 3

reload of lysates of rep 3

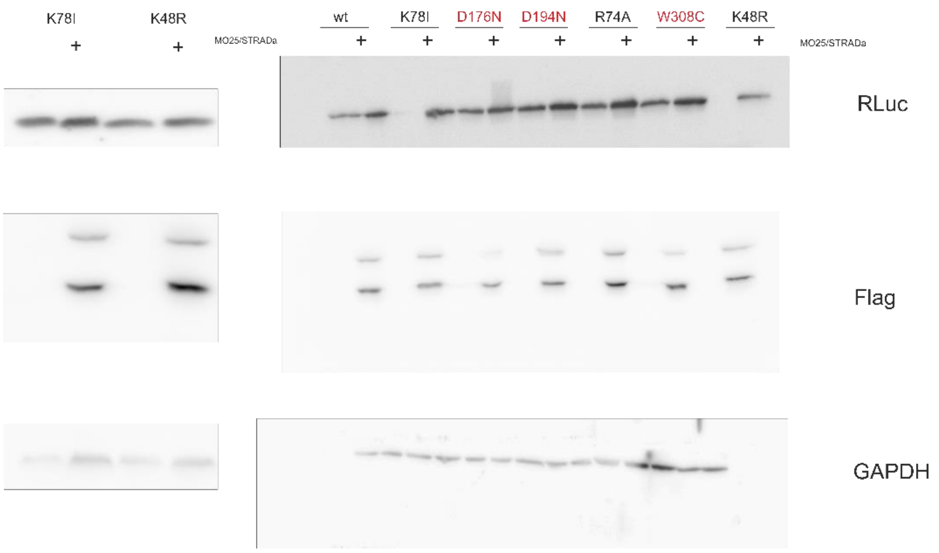

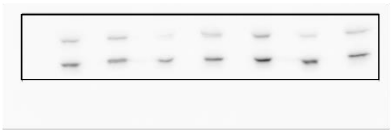

original western blot
Flag

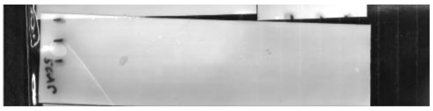

original marker picture
GAPDH

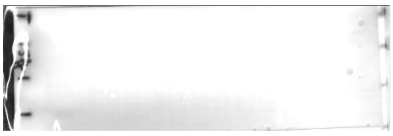

original marker picture
Flag

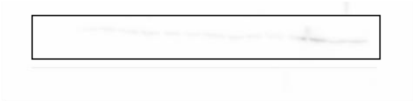

original western blot
GAPDH

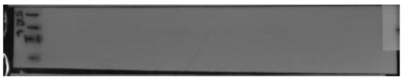

original marker picture
RLuc

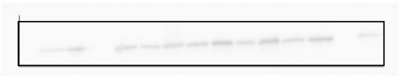

original western blot
RLuc

original marker picture

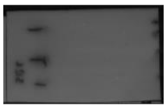

original western blot

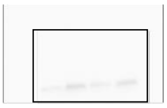

RLuc

original marker picture

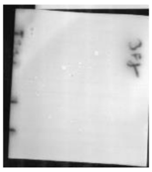

original western blot

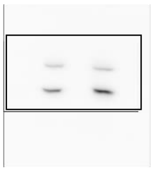

Flag

original marker picture

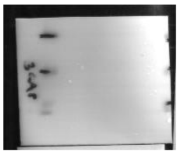

original western blot

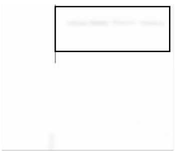

GAPDH

:

Replicate 4

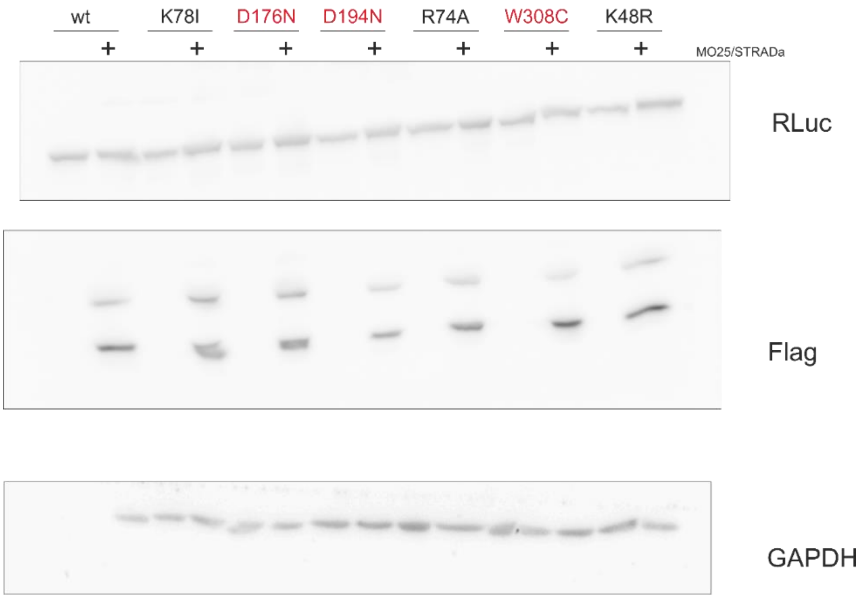

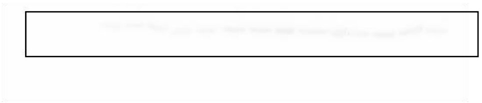

original western blot
GAPDH

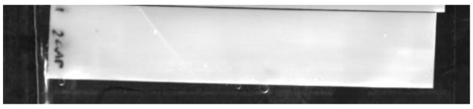

original marker picture
GAPDH

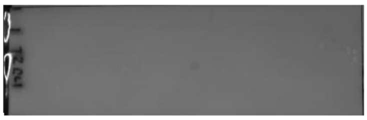

original marker picture
RLuc

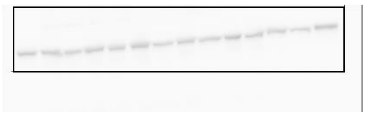

original western blot
RLuc

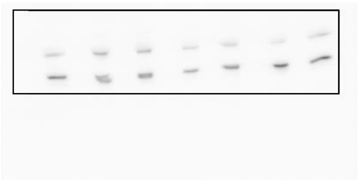

original western blot
Flag

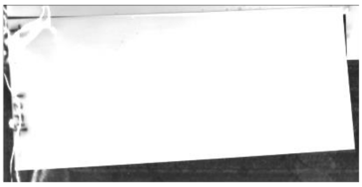

original marker picture
Flag

Replicate 5:

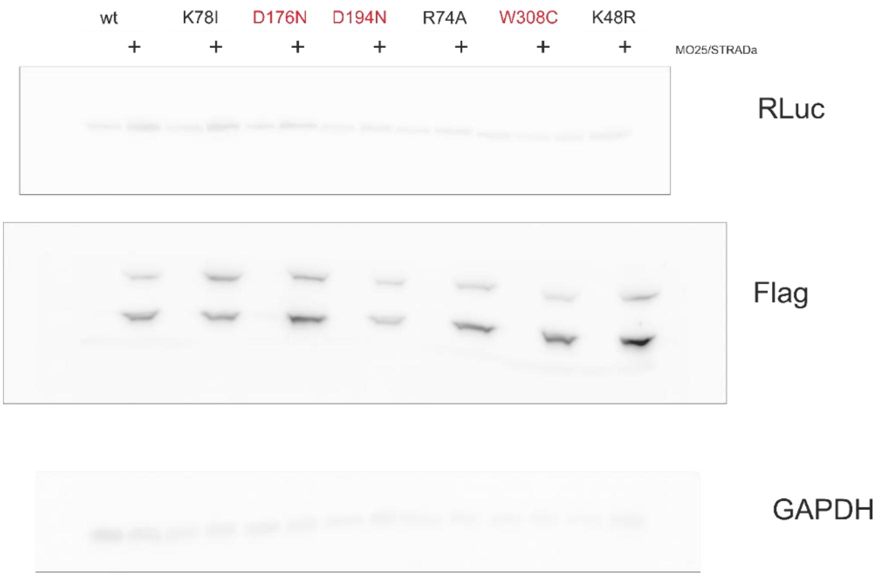

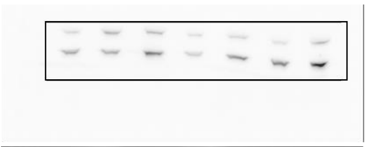

original western blot
Flag

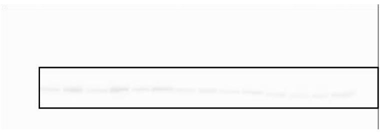

original western blot
RLuc

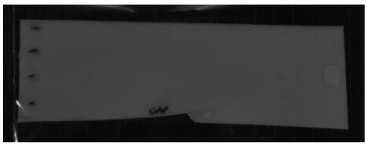

original marker picture
Flag/GAPDH

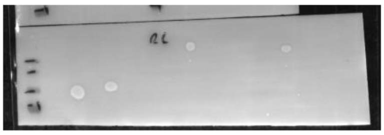

original marker picture
RLuc

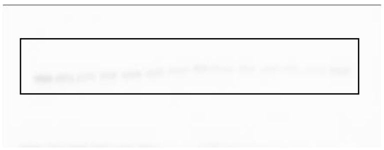

original western blot
GAPDH

No marker was detected for GAPDH in R5
