# Supplementary material for: Impact of protein and small molecule interactions on kinase conformations
Source: eLife. 2024 Aug 1;13:RP94755. doi: 10.7554/eLife.94755 (PMC11293870; doi:10.7554/eLife.94755)
Supplement: Figure 2—figure supplement 1—source data 2. [file elife-94755-fig2-figsupp1-data2.pdf]

Figure 2 – Figure Supplement 1: Complex formation of LKB1/STRADa/MO25 in HeLa and SW480 cells

In these panels representative western blots are shown. The corresponding western blot raw data is enclosed blow:

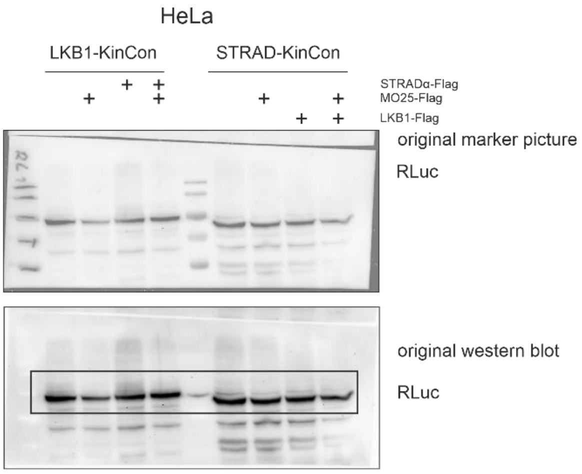

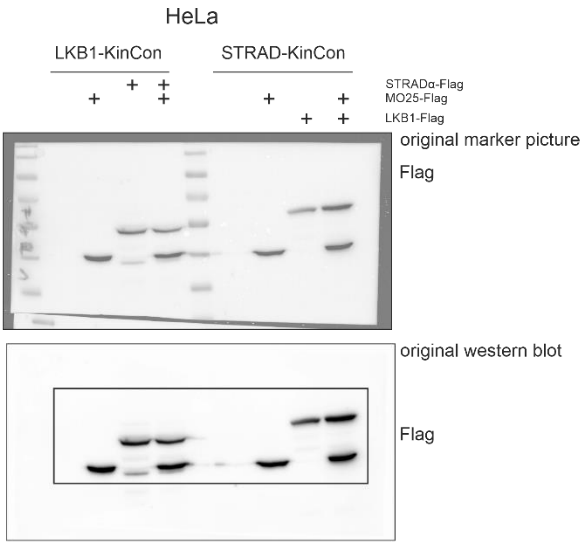

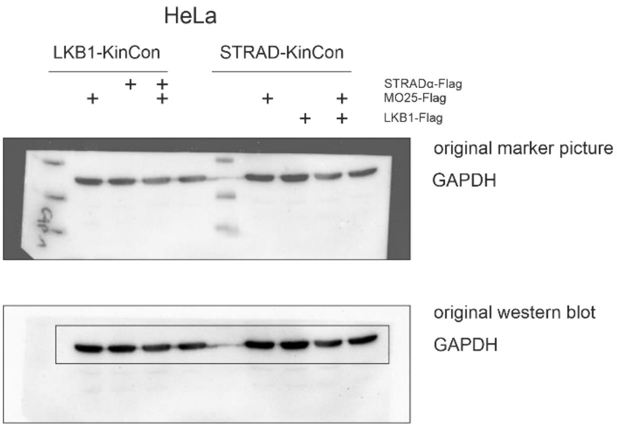

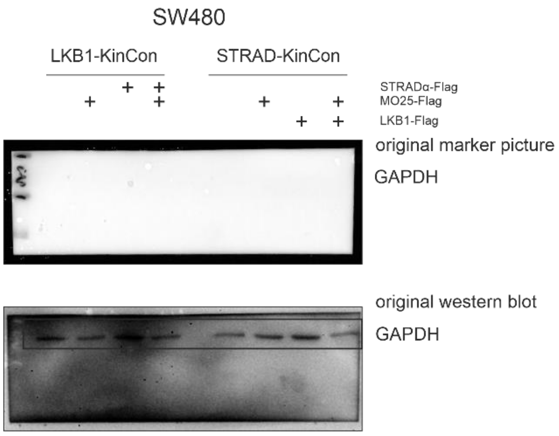
SW480
LKB1-KinCon
STRAD-KinCon
STRADα-Flag
MO25-Flag
LKB1-Flag
original marker picture
GAPDH
original western blot
GAPDH

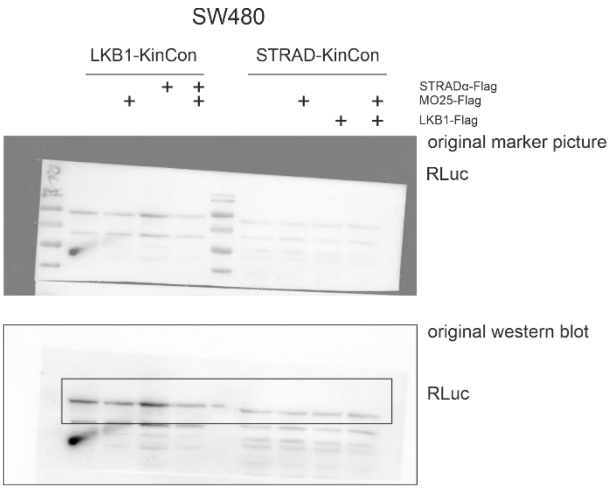
SW480
LKB1-KinCon
STRAD-KinCon
STRADα-Flag
MO25-Flag
LKB1-Flag
original marker picture
RLuc
original western blot
RLuc

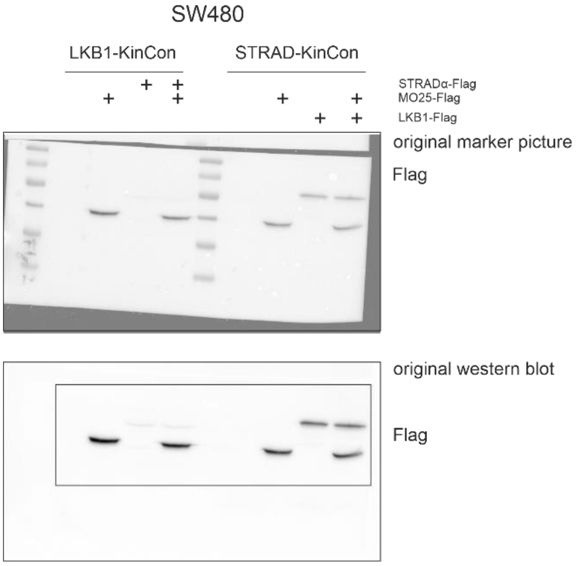
SW480
LKB1-KinCon
STRAD-KinCon
STRADα-Flag
MO25-Flag
LKB1-Flag
original marker picture
Flag
original western blot
Flag
