# Supplementary material for: Impact of protein and small molecule interactions on kinase conformations
Source: eLife. 2024 Aug 1;13:RP94755. doi: 10.7554/eLife.94755 (PMC11293870; doi:10.7554/eLife.94755)
Supplement: Figure 3—source data 2. [file elife-94755-fig3-data2.pdf]

Indicated antibodies have been used (for details see the Materials and Methods section)

Figure 3 panel D

TBZ treatment of RIPK1 and RIPK3 KinCon reporters.

The luciferase data was normalized on the western blots. One representative blot is shown in Figure 3 – Figure Supplement 1 panel A. The marked rectangles represent the used lanes.

Replicate 1:

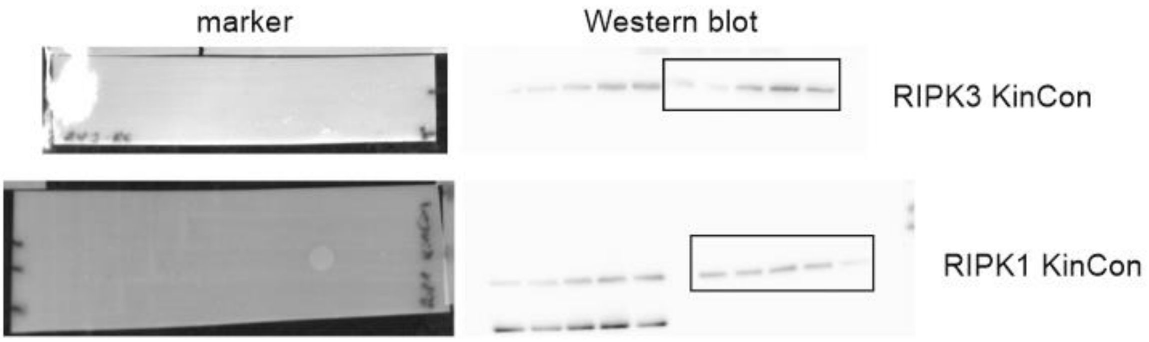

Replicate 2:

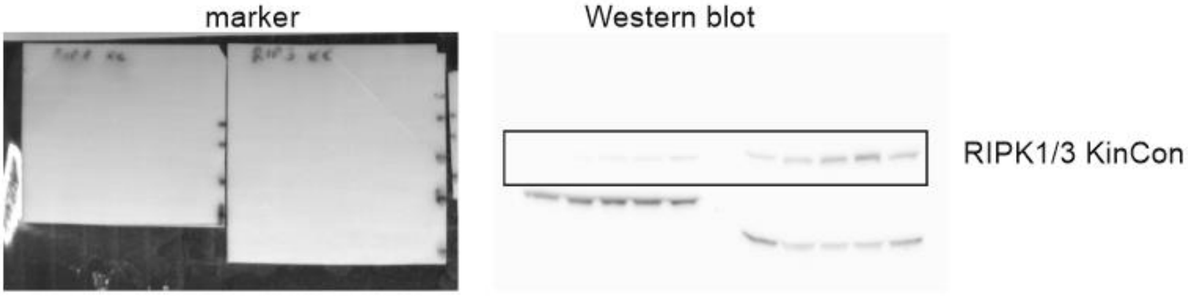

Replicate 3:

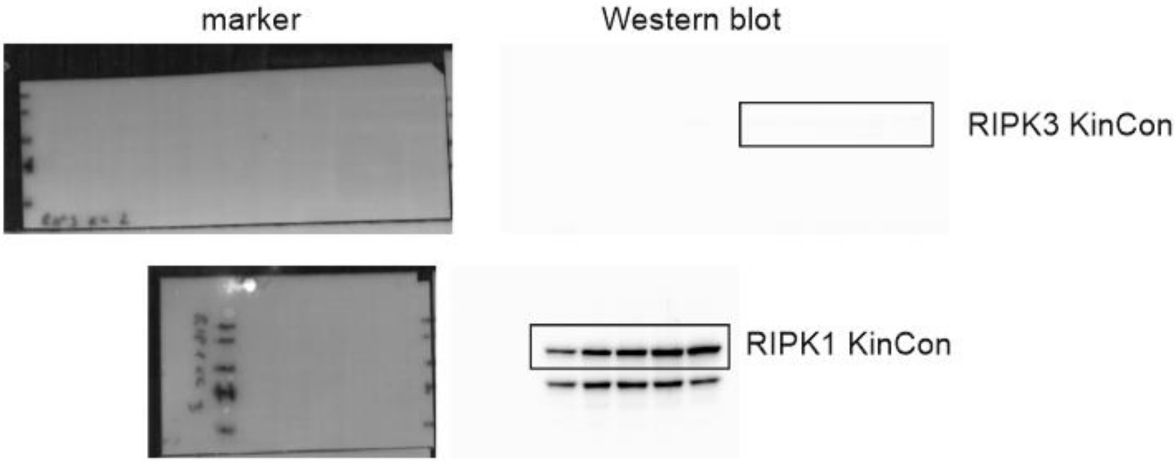

Figure 3 panel G + H

Basal signals of RIPK1 mutants.

The luciferase data was normalized on the western blots. More mutants were measured when the western blots were detected. The mutants depicted in the paper are highlighted in the western blots.

Replicate 1:

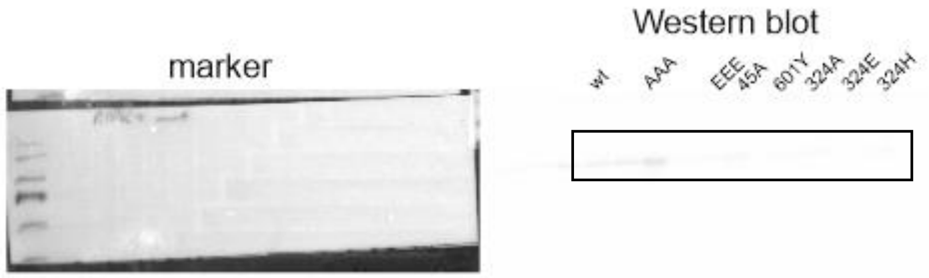

Replicate 2:

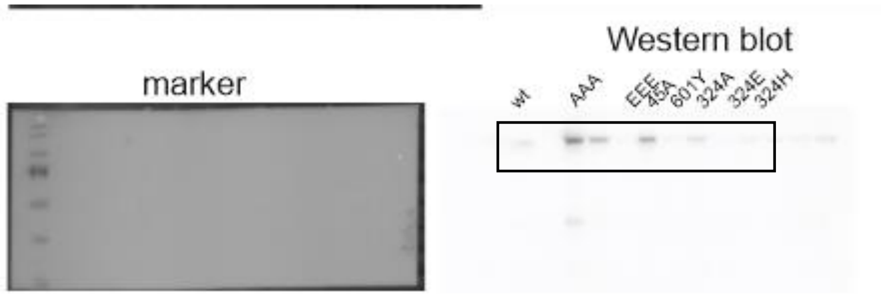

Replicate 3+4:

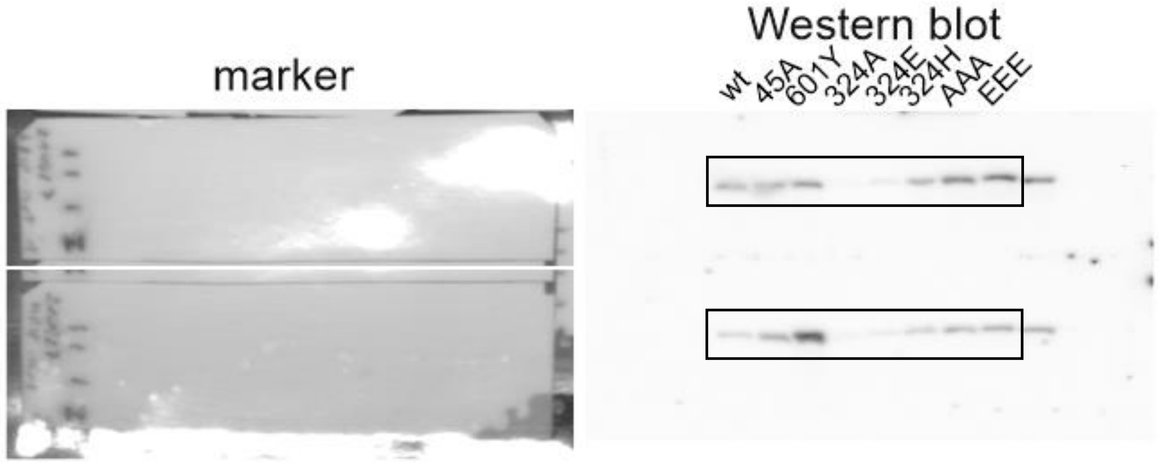

Replicate 5:

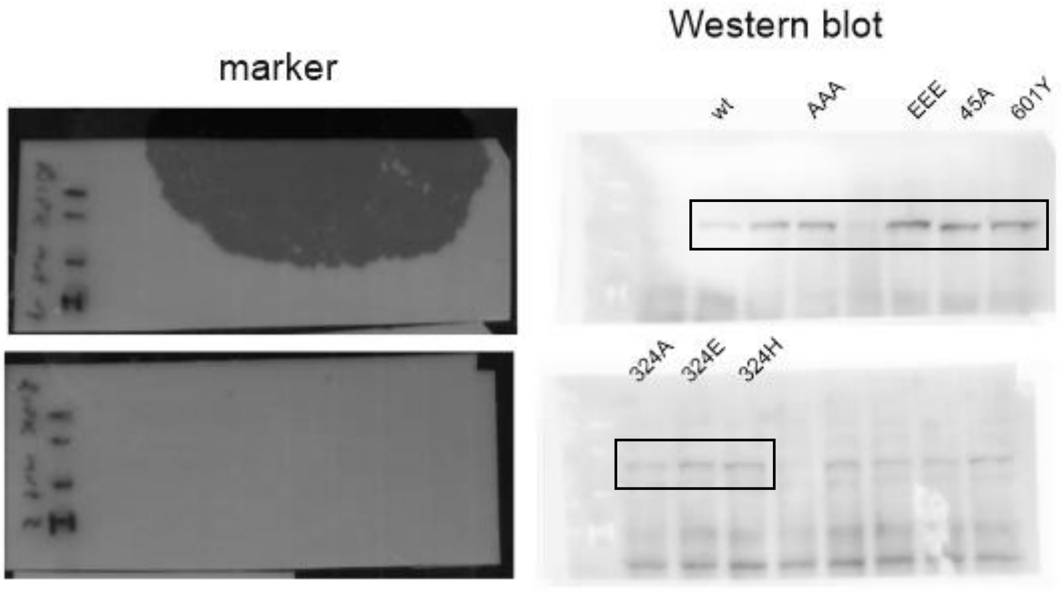
marker
Western blot
wt
AAA
EEE
45A
601Y
324A
324E
324H
