# Supplementary material for: Impact of protein and small molecule interactions on kinase conformations
Source: eLife. 2024 Aug 1;13:RP94755. doi: 10.7554/eLife.94755 (PMC11293870; doi:10.7554/eLife.94755)

Indicated antibodies have been used (for details see the Materials and Methods section)

Figure 3 – Figure Supplement 2 panel B

RIPK1 phosphorylation

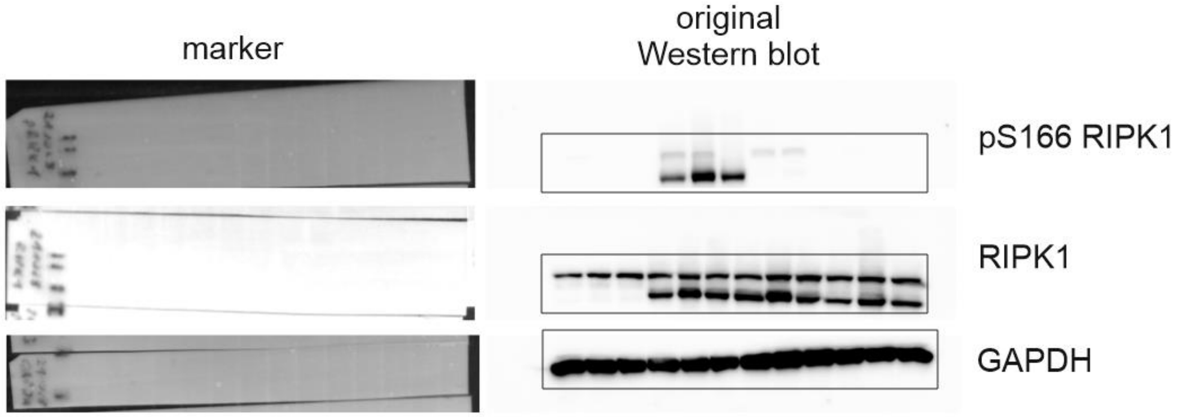

Supplement: Figure 3—figure supplement 2—source data 2. [file elife-94755-fig3-figsupp2-data2.pdf]
