# Supplementary material for: Impact of protein and small molecule interactions on kinase conformations
Source: eLife. 2024 Aug 1;13:RP94755. doi: 10.7554/eLife.94755 (PMC11293870; doi:10.7554/eLife.94755)

Indicated antibodies have been used (for details see the Materials and Methods section)

Figure 4 panel E

Basal signals of CDK4/6 KinCon.

In this panel one representative western blot is shown.

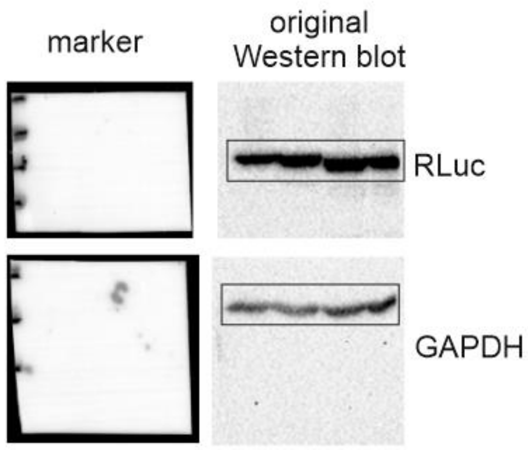

Figure 4 panel F

CDK4/6 KinCon reporters with inhibitor treatment.

In this panel one representative western blot is shown. The marked rectangles represent the shown lanes.

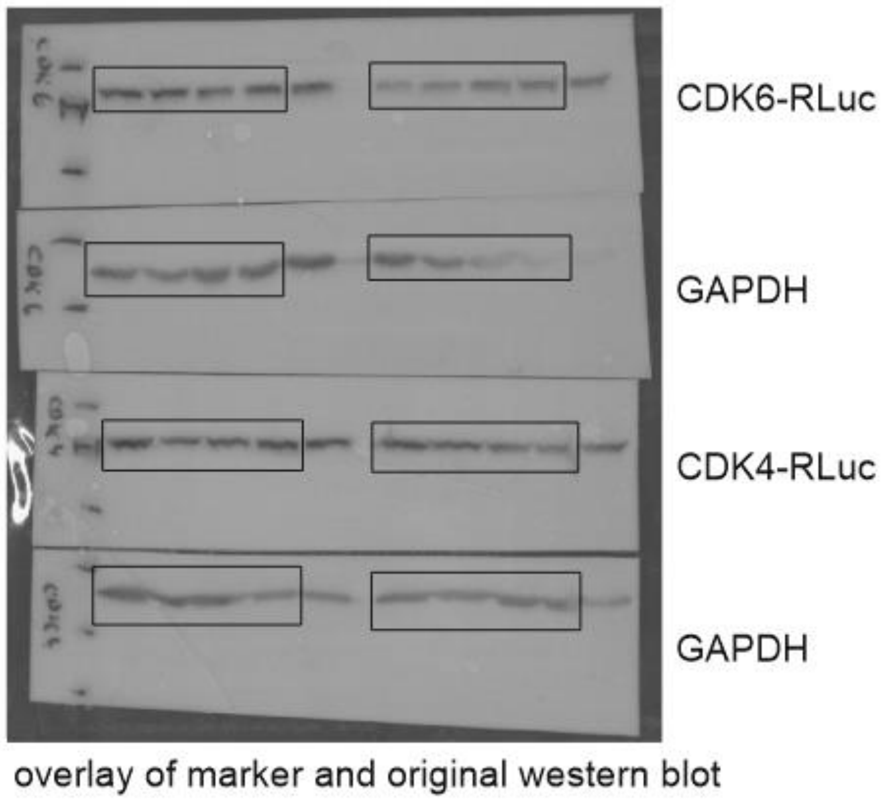

Supplement: Figure 4—source data 2. [file elife-94755-fig4-data2.pdf]
