# Supplementary figures and images for: Impact of protein and small molecule interactions on kinase conformations (part 4 of 4)
Source: eLife. 2024 Aug 1;13:RP94755. doi: 10.7554/eLife.94755 (PMC11293870; doi:10.7554/eLife.94755)

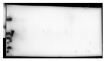

Supplement: Figure 5—figure supplement 1—source data 1. [file elife-94755-fig5-figsupp1-data1.zip › Figure 5 - Supplement 1/Replicate 3 marker Lamin PKAc.png]

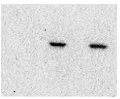

Supplement: Figure 5—figure supplement 1—source data 1. [file elife-94755-fig5-figsupp1-data1.zip › Figure 5 - Supplement 1/Replicate 2 WB GAPDH PKAc.png]

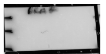

Supplement: Figure 5—figure supplement 1—source data 1. [file elife-94755-fig5-figsupp1-data1.zip › Figure 5 - Supplement 1/Replicate 1 marker RLuc BRAF.png]

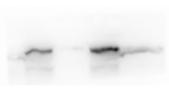

Supplement: Figure 5—figure supplement 1—source data 1. [file elife-94755-fig5-figsupp1-data1.zip › Figure 5 - Supplement 1/Replicate 3 WB RLuc BRAF.png]

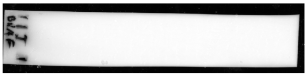

Supplement: Figure 5—figure supplement 1—source data 1. [file elife-94755-fig5-figsupp1-data1.zip › Figure 5 - Supplement 1/marker all Replicates BRAF.png]

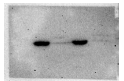

Supplement: Figure 5—figure supplement 1—source data 1. [file elife-94755-fig5-figsupp1-data1.zip › Figure 5 - Supplement 1/Replicate 3 WB GAPDH PKAc.png]

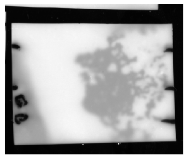

Supplement: Figure 5—figure supplement 1—source data 1. [file elife-94755-fig5-figsupp1-data1.zip › Figure 5 - Supplement 1/Replicate 2 marker GAPDH BRAF.png]

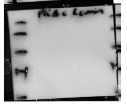

Supplement: Figure 5—figure supplement 1—source data 1. [file elife-94755-fig5-figsupp1-data1.zip › Figure 5 - Supplement 1/Replicate 1 marker Lamin PKAc.png]
