# Supplementary material for: Impact of protein and small molecule interactions on kinase conformations
Source: eLife. 2024 Aug 1;13:RP94755. doi: 10.7554/eLife.94755 (PMC11293870; doi:10.7554/eLife.94755)
Supplement: Figure 5—figure supplement 1—source data 2. [file elife-94755-fig5-figsupp1-data2.pdf]

Indicated antibodies have been used (for details see the Materials and Methods section)

Figure 5 – Figure Supplement 1 panel A:

Subcellular localization of PKAc and BRAF KinCon reporters.

N stands for nucleus and C stands for cytoplasm.

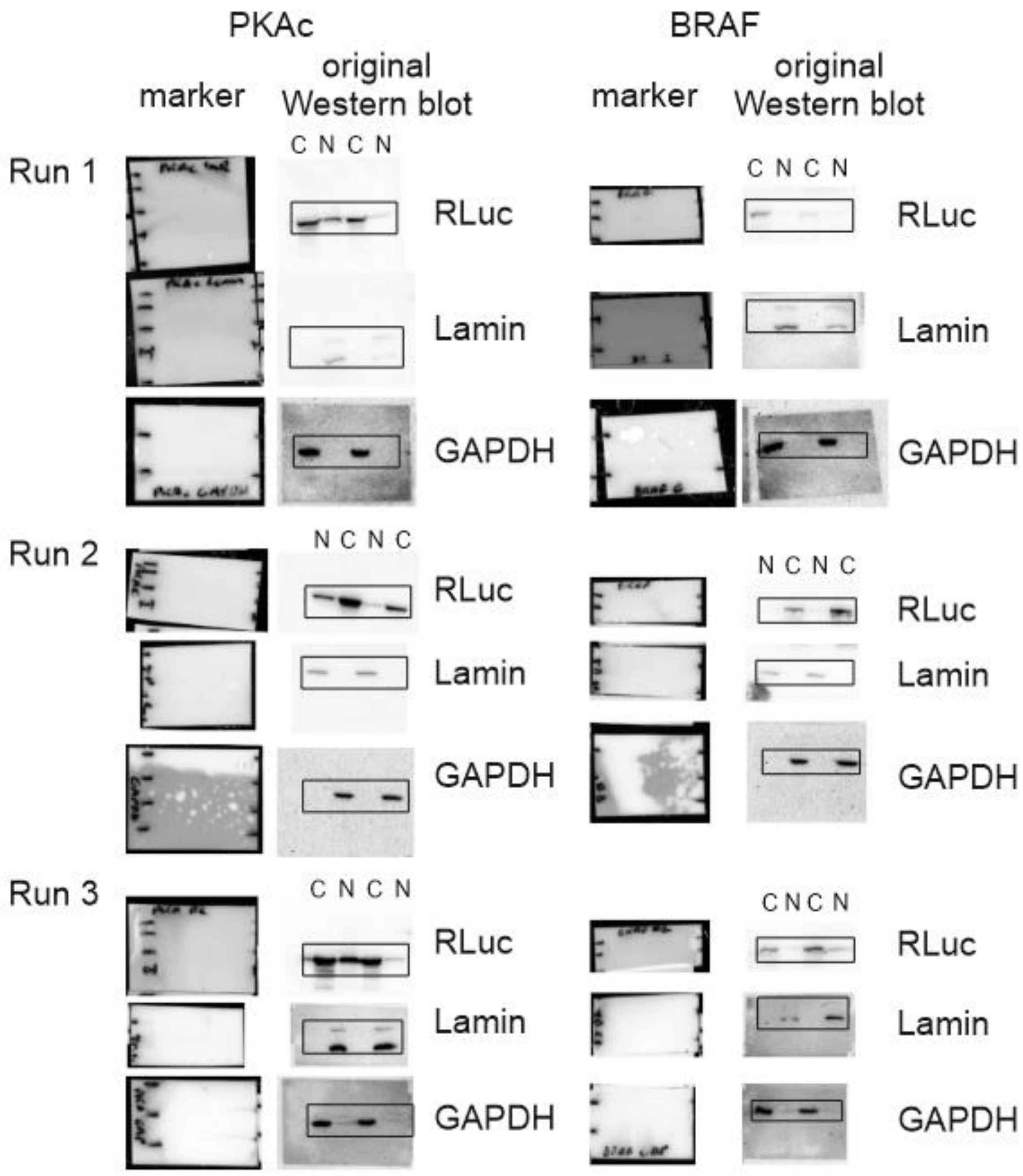

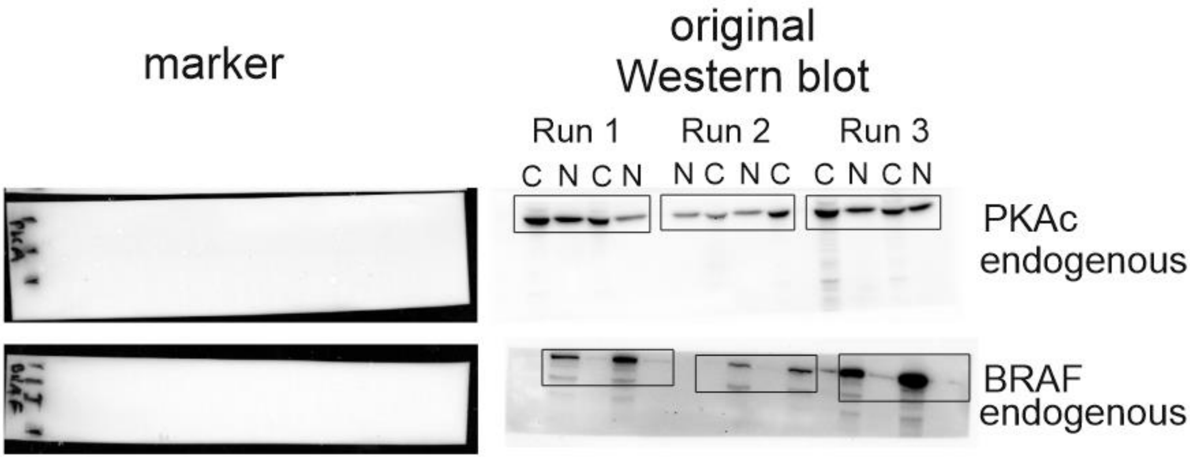
marker
original
Western blot
Run 1
Run 2
Run 3
C N C N
N C N C
C N C N
PKAc
endogenous
BRAF
endogenous
